# Supplementary material for: Genetic Architecture of Adaptive Immune System Identifies Key Immune Regulators
Source: Cell Rep. 2018 Oct 16;25(3):798–810.e6. doi: 10.1016/j.celrep.2018.09.048 (PMC6205839; doi:10.1016/j.celrep.2018.09.048)
Supplement: Document S1. Figures S1 and S2 [file mmc1.pdf]

Cell Reports, Volume *25*

Supplemental Information

Genetic Architecture

of Adaptive Immune System

Identifies Key Immune Regulators

Vasiliki Lagou, Josselyn E. Garcia-Perez, Ide Smets, Lies Van Horebeek, Marijne Vandebergh, Liye Chen, Klara Mallants, Teresa Prezzemolo, Kelly Hilven, Stephanie Humblet-Baron, Matthieu Moisse, Philip Van Damme, Guy Boeckxstaens, Paul Bowness, Bénédicte Dubois, James Dooley, Adrian Liston, and An Goris

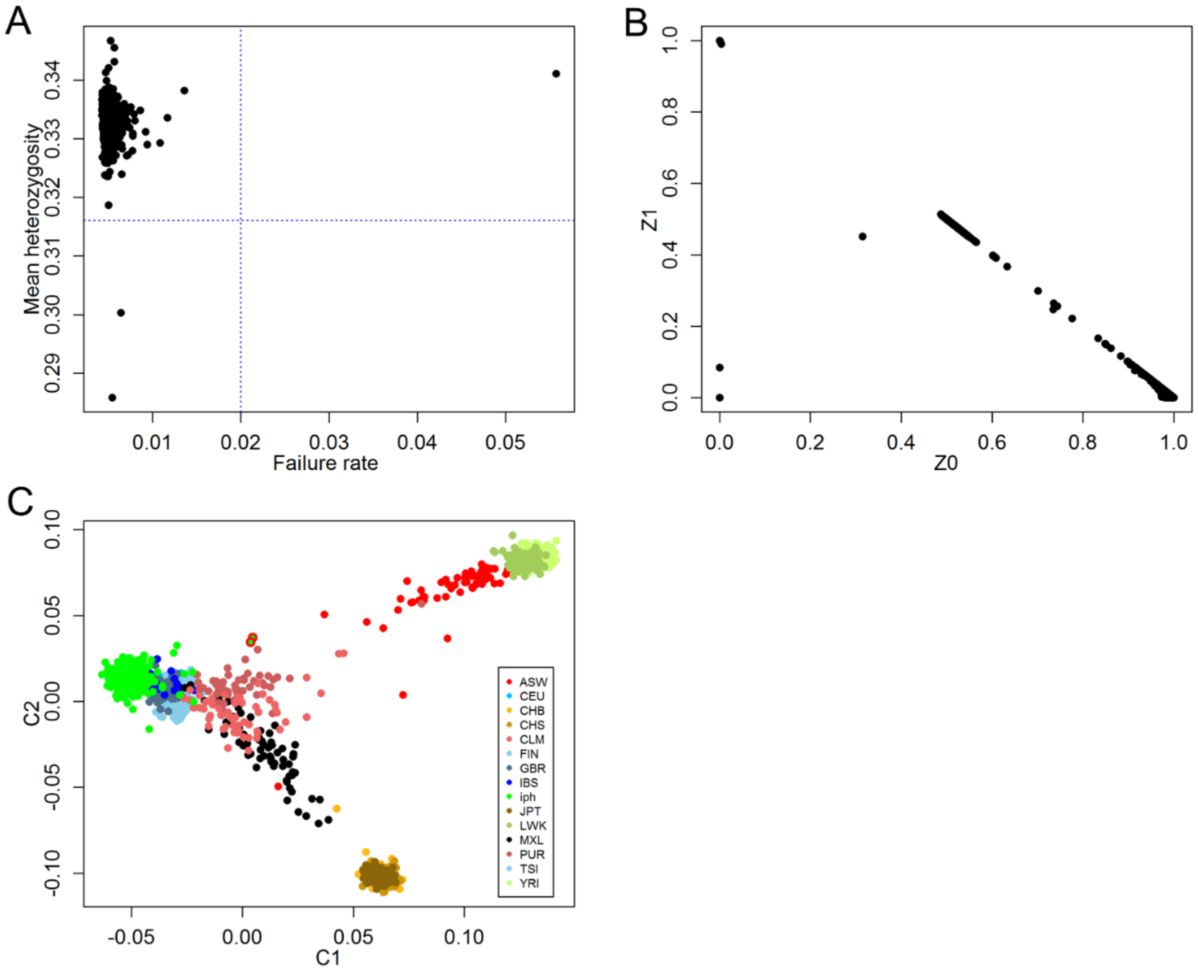

Figure S1. Sample quality control (QC) steps. Related to STAR Methods.
n = 502 samples were genotyped, and quality control (QC) was performed as described in the STAR Methods. n = 13 samples failed QC because of the following reasons: genotyping failure rate and heterozygosity (**A.,** n = 3), duplicates and relatedness (**B.**, n = 8) and ethnicity (**C.**, n = 2). n = 489 samples passed QC and were used as input for imputation as described in the STAR Methods.

A. Genotyping failure rate versus heterozygosity.
Sample QC was based on the set of variants meeting minor allele frequency (MAF) ≥ 5%, genotyping success rate ≥ 98% and Hardy-Weinberg P > 10^{-6}. Three samples with genotype call rate < 98% (1 sample) or excess heterozygosity (> 5 standard deviations from the mean) (2 samples) failed QC.

B. Identity-by-descent (IBD) analysis.
For the IBD analysis, we removed regions of extended linkage disequilibrium (LD) from the dataset and pruned remaining regions so that no pair of variants within a given window of 50 kb is correlated ($r^2 > 0.2$). The X- and Y- axis represent Z0 (P(IBD = 0)) and Z1 (P(IBD = 1)), the probabilities of sharing 0 or 1 alleles identical-by-descent. Four samples were identified as duplicates (expected Z0 = Z1 = 0), three samples as parent-offspring (Z0 = 0 and Z1 = 1), and one sample as half-sibling (expected Z0 = Z1 = 0.5).

C. Principal component analysis.
The first two principal components (C1 and C2) are calculated for the current dataset (Iph, bright green) together with 1000G Phase 1 data for the following populations: ASW = Americans of African Ancestry in SW USA; CEU = Utah Residents (CEPH) with Northern and Western European Ancestry; CHB = Han Chinese in Bejing, China; CHS = Southern Han Chinese; CLM = Colombians from Medellin, Colombia; FIN = Finnish in Finland; GBR = British in England and Scotland; IBS = Iberian Population in Spain; JPT = Japanese in Tokyo, Japan; LWK = Luhya in Webuye, Kenya; MXL = Mexican Ancestry from Los Angeles USA; PUR = Puerto Ricans from Puerto Rico; TSI = Toscani in Italia; YRI = Yoruba in Ibadan, Nigeria). Two individuals (green encircled with orange) were removed as outliers from the European cluster. Further inspection revealed these individuals as being of North-African descent.

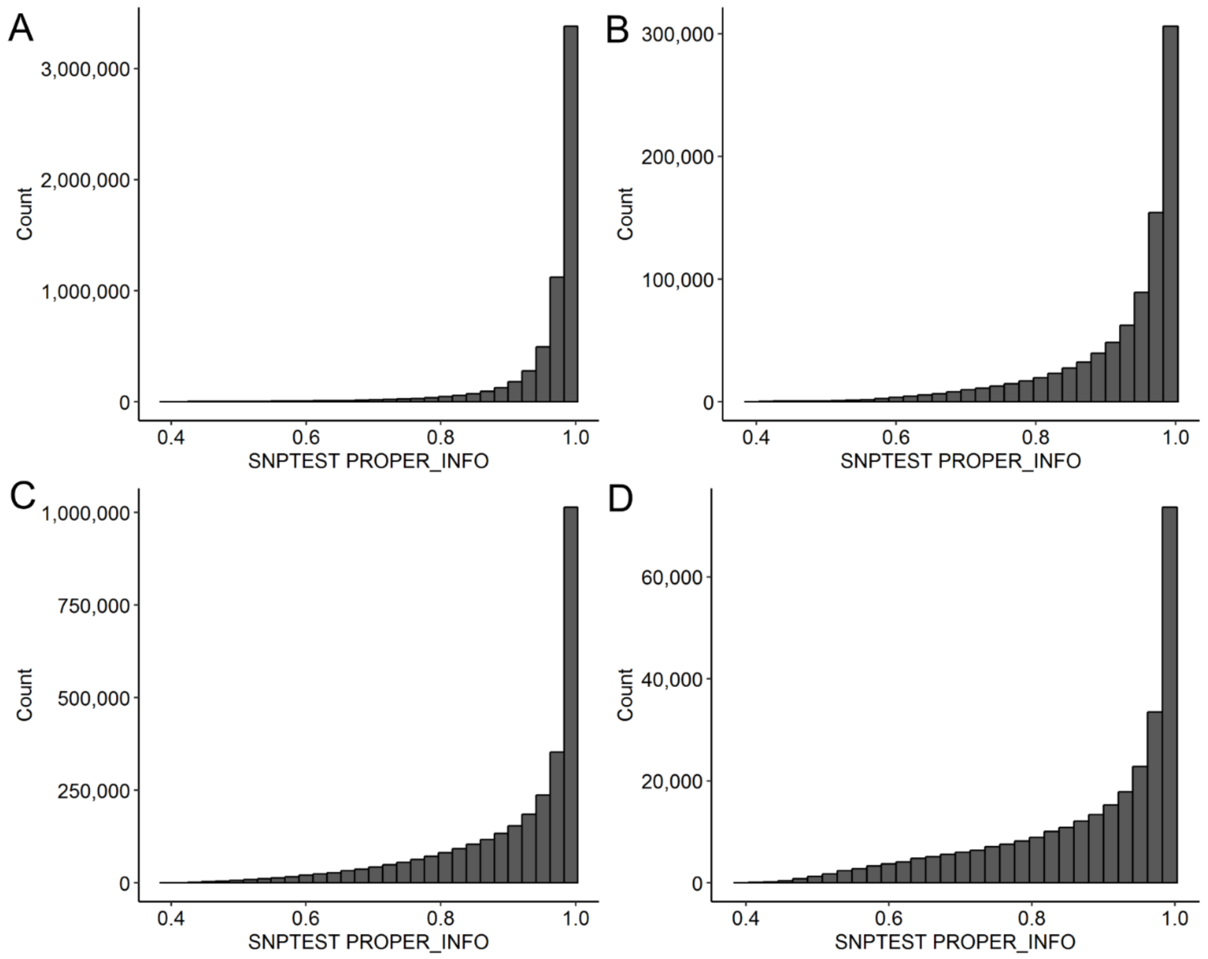

Figure S2. Imputation quality for genetic variants included upon QC. Related to STAR Methods. Histograms of the imputation quality scores (average SNPTEST PROPER_INFO across association results for n = 54 traits) for the following categories amongst 10,246,977 autosomal variants:

A. Common (MAF > 5%) single-nucleotide variants (n = 6,086,969)
B. Common (MAF > 5%) indels/structural variants (n = 907,465)
C. Less common (1% ≤ MAF ≤5%) single-nucleotide variants (n = 2,962,706)
D. Less common (1% ≤ MAF ≤ 5%) indels/structural variants (n = 289,837)
