# Supplementary material for: Genetic Architecture of Adaptive Immune System Identifies Key Immune Regulators
Source: Cell Rep. 2018 Oct 16;25(3):798–810.e6. doi: 10.1016/j.celrep.2018.09.048 (PMC6205839; doi:10.1016/j.celrep.2018.09.048)
Supplement: Document S2. Article plus Supplemental Information [file mmc8.pdf]

Cell Reports

Resource

Genetic Architecture of Adaptive Immune System Identifies Key Immune Regulators

Graphical Abstract

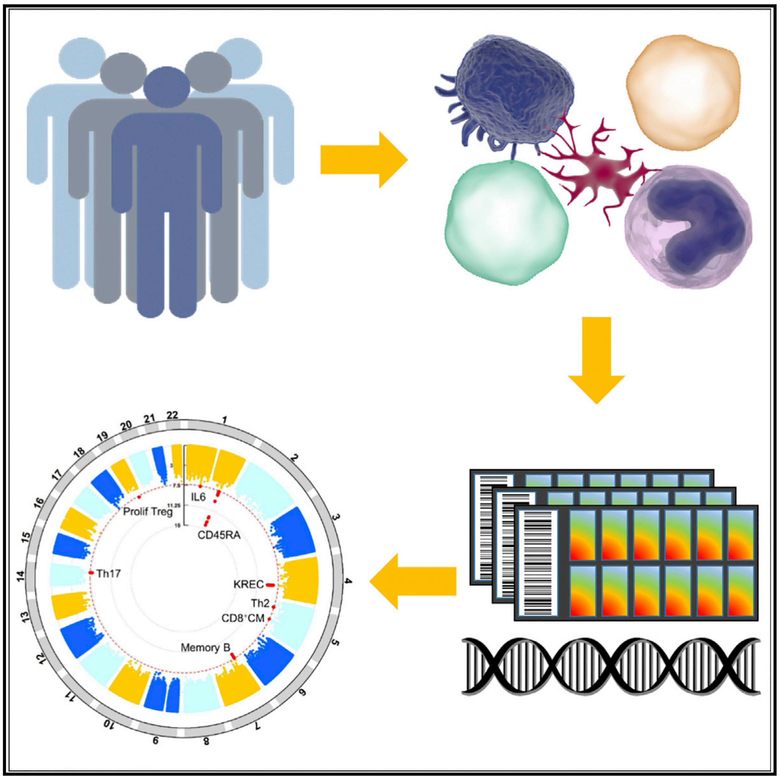

Authors

Vasiliki Lagou, Josselyn E. Garcia-Perez, Ide Smets, ..., James Dooley, Adrian Liston, An Goris

Correspondence

adrian.liston@vib-kuleuven.be (A.L.),
an.goris@kuleuven.be (A.G.)

In Brief

Lagou et al. identify genetic factors explaining interindividual variation in composition of the adaptive immune system. Factors pinpoint key human immune regulators controlling B and T cell differentiation and levels of disease-relevant T helper and regulatory cells. These findings shed light on mechanisms of autoimmune disease and offer therapeutic perspectives.

Highlights

- GWAS provides understanding of genetic factors shaping adaptive immune system
- Common and less common variants explain 10% of variance in cellular variables
- Associations pinpoint key regulators of B and T cell differentiation
- Associations offer therapeutic targets for controlling pro-inflammatory traits

Lagou et al., 2018, Cell Reports *25*, 798–810
October 16, 2018 © 2018 The Authors.
https://doi.org/10.1016/j.celrep.2018.09.048

CellPress

OPEN ACCESS
CellPress

Cell Reports
Resource

Genetic Architecture of Adaptive Immune System Identifies Key Immune Regulators

Vasiliki Lagou,[1,2,3,4] Josselyn E. Garcia-Perez,[2,3,4] Ide Smets,[1,4,5] Lies Van Horebeek,[1,4] Marijne Vandebergh,[1,4] Liye Chen,[6] Klara Mallants,[1,4] Teresa Prezzemolo,[2,3,4] Kelly Hilven,[1,4] Stephanie Humblet-Baron,[2,3,4] Matthieu Moisse,[4,7,8] Philip Van Damme,[4,5,7,8] Guy Boeckxstaens,[9,10] Paul Bowness,[6] Bénédicte Dubois,[1,4,5] James Dooley,[2,3,4] Adrian Liston,[2,3,4,11,*] and An Goris[1,4,11,12,*]
[1]KU Leuven Department of Neurosciences, Laboratory for Neuroimmunology, 3000 Leuven, Belgium
[2]VIB Center for Brain & Disease Research, Laboratory for Translational Immunology, 3000 Leuven, Belgium
[3]KU Leuven Department of Immunology and Microbiology, Laboratory for Translational Immunology, 3000 Leuven, Belgium
[4]Leuven Brain Institute (LBI), Leuven, Belgium
[5]Department of Neurology, University Hospitals Leuven, 3000 Leuven, Belgium
[6]Botnar Research Centre, Nuffield Department of Orthopaedics, Rheumatology and Musculoskeletal Sciences, University of Oxford, Oxford OX3 7LD, UK
[7]VIB Center for Brain & Disease Research, Laboratory of Neurobiology, 3000 Leuven, Belgium
[8]KU Leuven Department of Neurosciences, Experimental Neurology, 3000 Leuven, Belgium
[9]KU Leuven Department of Chronic Diseases, Metabolism and Ageing, Translational Research Center for GI Disorders (TARGID), 3000 Leuven, Belgium
[10]Department of Gastroenterology, University Hospitals Leuven, 3000 Leuven, Belgium
[11]These authors contributed equally
[12]Lead Contact
*Correspondence: adrian.liston@vib-kuleuven.be (A.L.), an.goris@kuleuven.be (A.G.)
https://doi.org/10.1016/j.celrep.2018.09.048

SUMMARY

The immune system is highly diverse, but characterization of its genetic architecture has lagged behind the vast progress made by genome-wide association studies (GWASs) of emergent diseases. Our GWAS for 54 functionally relevant phenotypes of the adaptive immune system in 489 healthy individuals identifies eight genome-wide significant associations explaining 6%–20% of variance. Coding and splicing variants in *PTPRC* and *COMMD10* are involved in memory T cell differentiation. Genetic variation controlling disease-relevant T helper cell subsets includes *RICTOR* and *STON2* associated with Th2 and Th17, respectively, and the interferon-lambda locus controlling regulatory T cell proliferation. Early and memory B cell differentiation stages are associated with variation in *LARP1B* and *SP4.* Finally, the latrophilin family member *ADGRL2* correlates with baseline pro-inflammatory interleukin-6 levels. Suggestive associations reveal mechanisms of autoimmune disease associations, in particular related to pro-inflammatory cytokine production. Pinpointing these key human immune regulators offers attractive therapeutic perspectives.

INTRODUCTION

The immune system is characterized by enriched polymorphism in genetic control factors, coupled to a high degree of cellular plasticity and sensitivity to environmental drivers. The resulting functional diversity serves as an important control mechanism for limiting the impact of transmissible pathogens on the population. Conversely, this same diversity contributes to the susceptibility or resistance of individuals to a broad set of sterile diseases, from those with an obvious immunological component, such as autoimmunity, allergy, inflammation, and cancer, to the increasingly recognized immune-influenced diseases, such as cardiovascular, metabolic, and neurological diseases. Despite this, the characterization of the genotype-phenotype relationship of the immune system components has lagged behind the vast progress made by genome-wide association studies (GWASs) of emergent diseases.

The recent advent of in-depth immune phenotyping across large sample sizes has enabled characterization of the extent and identification of the factors shaping variation in the human immune profile (Liston et al., 2016). Longitudinal studies have reported a high level of interindividual variation, with low longitudinal variation and a highly elastic structure, where transient antigen-induced changes are followed by a return to the individual's unique baseline (Carr et al., 2016; Orrù et al., 2013; Tsang et al., 2014). Twin and family-based studies provide heritability estimates of 20%–40% on average but cover a wide range across individual cellular or cytokine traits (Brodin et al., 2015; Carr et al., 2016; Mangino et al., 2017; Orrù et al., 2013; Roederer et al., 2015). Aging contributes up to 5% of total immune variation (Aguirre-Gamboa et al., 2016; Brodin et al., 2015; Carr et al., 2016; Orrù et al., 2013; Patin et al., 2018; Shen-Orr et al., 2016), and environmental factors shaping the immune system include obesity, cohabitation, and chronic viral infections

This is an open access article under the CC BY license (http://creativecommons.org/licenses/by/4.0/).

Table 1. Replication of Previously Known Genotype-Immune Phenotype Associations

Chr	Pos	rsID	EA	NEA	EAF	BETA (SE)	p Value	Trait	Candidate Genes	Trait (Orrù et al., 2013)
2	38897074	rs13011383	G	A	0.87	−0.34 (0.12)	.0070	$CD4^+$ EMRA	*GALM*, *HNRPLL*	TD $CD4^+$ %GP
2	38921934	rs7583259	G	C	0.49	−0.31 (0.09)	.00051	$CD8^+$ EM	*GALM*, *DHX57*, *HNRPLL*	$CD45RA^-$ $CD28^-$ $CD8^{br}$ %P
2	87014377	rs2944254	C	T	0.72	0.25 (0.09)	.0073	$CD4^+$ proliferating	*CD8A*, *RMND5A*, *CD8B*, *VPS24*	$CD4^+$ $CD8^{dim}$ AC
12	6899181	rs2855537	G	T	0.76	0.18 (0.08)	.023	TREC	*CD4*	naive ($CD4^+$ $CD8^+$) AC
17	33797371	rs9916257	T	G	0.47	−0.23 (0.06)	.00028	NK	*SLFN13*, *SLFN12L*, *CCL1*	NK %GP

We observed nominal significance for five genome-wide significant associations previously reported in the Sardinian population (Orrù et al., 2013). Trait names in Orrù et al. study: AC, absolute count; %GP, percentage of grandparental cells; NK, natural killer (cells); %P, percentage of parental cells; TD, terminally differentiated. BETA, effect; Chr, chromosome; EA, effect allele; EAF, effect allele frequency; NEA, non-effect allele; Pos, position in GRCh37; rsID, reference SNP identification; SE, standard error.

(Aguirre-Gamboa et al., 2016; Brodin et al., 2015; Carr et al., 2016; Patin et al., 2018).

Identification of the genetic factors controlling variation in the immune system is still in the initial discovery phase, reminiscent of the early days of disease-susceptibility GWASs, with novel and strong associations emerging from the pioneer studies (Aguirre-Gamboa et al., 2016; Orrù et al., 2013; Patin et al., 2018; Roederer et al., 2015), but the overlap of loci reported in more than one study is still limited (Liston and Goris, 2018). Hence, we undertook a GWAS for 54 immune traits enriched for functionally relevant adaptive immune system phenotypes, including 30 T cell and 8 B cell subsets based on proliferation, differentiation, activation, or cytokine production, as well as baseline *ex vivo* plasma levels of ten pro- or anti-inflammatory cytokines. Our GWAS covers the genetic contributions from both common (>5%) and less common (1%–5%) variants. Genome-wide significant associations explain a median of 10% of variance in adaptive immune system variation and identify variant genes and pathways as key regulators of the adaptive immune system in humans. Coding and splicing variants in *PTPRC* and *COMMD10* are involved in memory T cell differentiation. Genetic variation controlling T helper cell subsets with crucial roles in protection against infection and susceptibility to autoimmune disease include the second mTOR signaling complex (*RICTOR*) and endocytosis-related stonin 2 (*STON2*) associated with Th2 and Th17, respectively, and the interferon-lambda locus controlling regulatory T (Treg) cell proliferation. Early and memory B cell differentiation stages are associated with variation in the as yet poorly characterized genes *LARP1B* and *SP4*. Finally, our results implicate the latrophilin family member *ADGRL2* as genetic variant for baseline pro-inflammatory cytokine production. Our data furthermore unravel the mechanism of action of established genotype-disease associations, involving key cytokines such as tumor necrosis factor alpha (TNF-α) and interleukin-2 (IL-2) in autoimmune diseases and granulocyte-macrophage colony-stimulating factor (GM-CSF) in immune-proliferative diseases. Finally, clinical implications resulting from associations in this study offer attractive therapeutic intervention points.

RESULTS

A Genome-Wide Association Screen for Common and Less Common Variants Controlling the Human Adaptive Immune System

We performed a GWAS in a study population of 502 healthy white individuals for 54 immune phenotypes. Immune phenotypes were enriched for functionally relevant adaptive immune system parameters and included 42 cellular phenotypes determined by flow cytometry and ten cytokines measured in plasma as described previously (Carr et al., 2016), as well as two DNA markers reflecting newly formed B and T cells (excision circles sjKREC [kappa-deleting recombination excision circle] and sjTREC [T cell receptor excision circle]) (van Zelm et al., 2011) (Table S1). We previously demonstrated stability over time for cellular immune variables in a subset of 177 individuals from this dataset who were sampled at multiple time points with an average of 6 months between samplings (Carr et al., 2016). The latest-generation imputation-based genotyping array allowed investigation of up to 10,246,977 autosomal variants with imputation accuracy (INFO) $\geq$ 0.4, including 6,994,434 common (minor allele frequency [MAF] >5%) and 3,252,543 less common (1 $\leq$ MAF $\leq$ 5%) variants in 489 individuals after quality control (QC) (Figures S1 and S2).

We observed nominal significance for five genome-wide significant associations previously reported in the Sardinian population (Orrù et al., 2013) (Table 1). Replication of previously known loci demonstrates the reproducibility of our dataset, despite different ethnic composition, different definitions for immunological variables, and independent generation of immune phenotyping platforms.

Subsequently, we identified eight regions reaching genome-wide significance ($p < 5 \times 10^{-8}$) to at least one immunological parameter (Figure 1); all of them were not previously reported. Lead variants in these regions had MAFs between 2% and 37% and explained 6.22% to 20.08% of the variance in the corresponding trait (Table 2). For all three regions where both trait and variant have appropriate equivalents in three previous GWASs (Aguirre-Gamboa et al., 2016; Orrù et al., 2013;

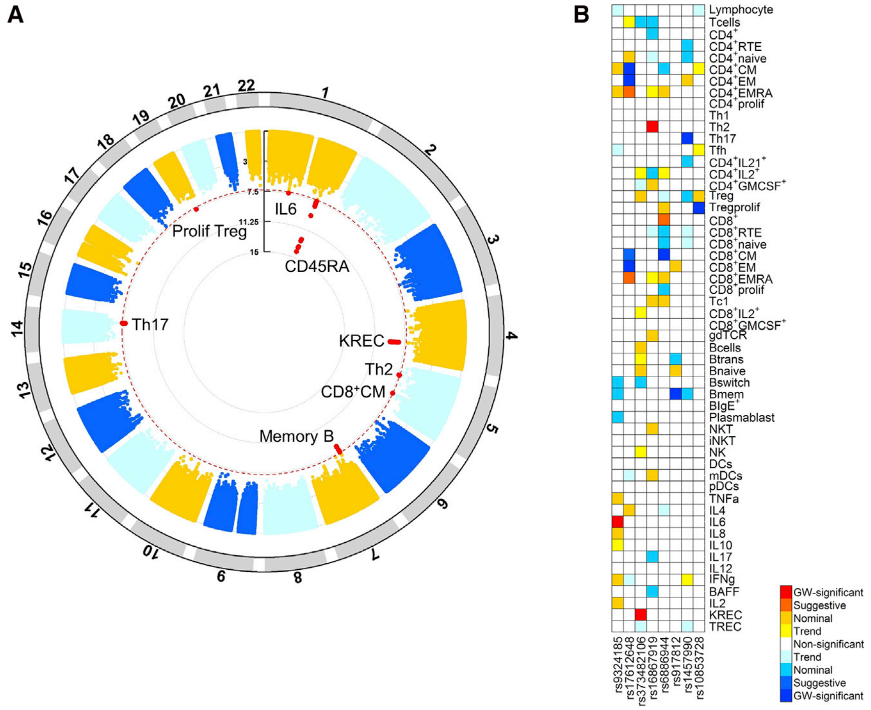

Figure 1. Genome-wide Significant Genotype-Immune Phenotype Associations

(A) Circos plot demonstrating eight regions reaching genome-wide significant association with immune phenotypes. The y axis displays the negative logarithm of the p value. Variants reaching genome-wide significance ($p < 5 \times 10^{-8}$, dotted red line) are depicted in red, and the corresponding trait with which the variant is associated is indicated.

(B) Overview of the association of eight independent lead variants reaching genome-wide significance to at least one immune phenotype with all 54 immune phenotypes (see also Table S1 for definitions of immune phenotypes). Darkest colors indicate genome-wide significant associations, whereas red and blue colors distinguish a positive or negative direction of effect, respectively. Genome-wide (GW) significant, suggestive, nominal, and trend correspond to p values $< 5 \times 10^{-8}$, $< 1 \times 10^{-4}$, < 0.05, and < 0.10, respectively.

Roederer et al., 2015), our findings replicated with nominal significance ($p < 0.05$) or showed a trend in the same direction in publicly available data (Table S2). Of note, the *PTPRC* variant with an MAF 2% fell beyond the scope of common variants in previous GWASs but is identified in our study covering the entire range of common and less common variants. For the other five genome-wide significant regions, the same trait and immunological definition was not investigated in previous GWASs.

Through a combination of bioinformatics and experimental functional analyses, the biologically most likely candidate gene stood out for all eight genome-wide significant associations. These associations highlight genes with critical roles in the adaptive immune system that have previously been demonstrated in mice but for which human data were lacking so far. Additionally, they shed light on the role of recently described but still poorly characterized protein families. Finally, they have important clinical implications.

Coding and Splicing Variants Involved in T Cell Memory Differentiation

Two of the genome-wide hits correspond to or are in high linkage disequilibrium (LD) with splicing or coding variants (Figure 2). The single-nucleotide polymorphisms (SNPs) rs17612648 and rs113116201 are in LD ($r^2 = 0.62$ in Europeans [EURs], $r^2 = 1$ in Utah Residents [CEPH] with Northern and Western ancestry [CEU]), and conditional analyses were not able to distinguish between them (Table S3). However, rs17612648 is a synonymous variant (P59P) located in exon 4 of *PTPRC*, the gene encoding protein-tyrosine phosphatase receptor-type C or CD45. Its minor allele (frequency = 2%) disrupts an exonic splicing silencer and increases levels of the splice form including exon 4 (CD45RA) in T cell lines (Jacobsen et al., 2000; Lynch and Weiss, 2001; Schwinzer and Wonigeit, 1990; Zhang et al., 2015b). The negative association of this allele with relative percentages of CD45RA$^-$ effector memory (EM) and central memory (CM) T cells, and positive association with CD45RA$^+$ terminally differentiated memory (EMRA) T cells, reflects persistent isoform expression on the cell surface as a marker for these cells (Figures 2A–2H).

Variant rs6886944, associated with CM CD8$^+$ T cells, is in near-perfect LD ($r^2 = 0.96$) with synonymous variant rs1129494 (T68T) in the fourth exon of the nearest gene, *COMMD10*, a member of the copper metabolism gene MURR1-domain-containing family (Figures 2I and 2J). These variants overlap with an expression quantitative trait locus (eQTL) for *COMMD10* in lymphoblastoid cell lines (LCL) and monocytes (Liang et al., 2013; Zeller et al., 2010) and correspond to the peak of association with exon-level expression of *COMMD10* exon 4 in LCL (r^2 with top-associated exon-level eQTL = 0.99) (Lappalainen et al., 2013).

RICTOR, *STON2*, and Lambda Interferons Drive T Helper Differentiation and Proliferation

Naive CD4$^+$ T cells can differentiate into functionally distinct subsets characterized by a unique cytokine expression pattern and a lineage-associated transcription factor network. The crucial role of these T helper subsets in infection, autoimmunity, and cancer has been demonstrated extensively. Our previous work indicates that in healthy individuals, baseline T helper differentiation is variant between individuals but stable over time (Carr et al., 2016). Our current study reports three genetic variants associated with T helper cell differentiation and activation (Figure 3).

Variant rs16867919 is located in an intron of the gene encoding *RICTOR*, part of the second mTOR signaling complex (mTORC2). This variant is predicted to disrupt the binding site

Table 2. Novel Genome-wide Significant Genotype-Immune Phenotype Associations

Chr	Pos	rsID	EA	NEA	EAF	BETA (SE)	% var	INFO	n	p value	Trait	Gene	Annotation
1	82196322	rs9324185	C	T	0.37	0.36 (0.06)	6.22	0.98	474	3.51×10^{-8}	IL-6	*ADGRL2*	G
1	198665917	rs17612648	G	C	0.02	−2.04 (0.26)	15.70	0.883	362	6.22×10^{-15}	$CD4^+$ EM	*PTPRC*	Co, Q, G, L
1	198665917	rs17612648	G	C	0.02	−1.76 (0.27)	11.84	0.859	362	8.94×10^{-11}	$CD4^+$ CM	*PTPRC*	Co, Q, G, L
1	198830942	rs113116201	C	T	0.02	1.72 (0.29)	12.26	0.996	255	7.58×10^{-9}	$CD4^+$ EMRA	*PTPRC*	Co, Q, L
1	198830942	rs113116201	C	T	0.02	−2.17 (0.27)	20.08	0.996	248	7.52×10^{-14}	$CD8^+$ EM	*PTPRC*	Co, Q, L
4	128924522	rs373482106	C	CA	0.23	0.49 (0.08)	8.21	0.919	449	4.90×10^{-10}	KREC	*LARP1B*	Q, L, E
5	38974929	rs16867919	G	A	0.21	0.43 (0.08)	6.13	1	475	3.56×10^{-8}	Th2	*RICTOR*	R, G, L
5	115413042	rs6886944	T	C	0.67	−0.54 (0.10)	11.57	0.966	248	4.83×10^{-8}	$CD8^+$ CM	*COMMD10*	Co, Q, Cs, N, L
7	21115110	rs917812	G	C	0.22	−0.46 (0.08)	6.87	0.967	468	5.86×10^{-9}	Memory B	*SP4*	Cs, L, E
14	82778603	rs1457990	A	G	0.51	−0.36 (0.06)	6.56	0.998	466	1.91×10^{-8}	Th17	*STON2*	Cs, L, E
19	39745146	rs10853728	G	C	0.67	−0.48 (0.08)	9.95	1	294	2.89×10^{-8}	proliferating Treg	IFNλ cluster	N, L

Variants associated with $p < 5 \times 10^{-8}$. SNPs rs17612648 and rs113116201 at the *PTPRC* locus are in LD ($r^2 = 0.62$ in EURs, $r^2 = 1$ in CEU) and conditional analyses were not able to distinguish between them (see also Table S3). Annotation indicates whether the variant is or is in LD with ($r^2 > 0.8$) a coding variant (Co) or known splicing or expression quantitative trait locus (Q) or is conserved (Cs), whether the variant disrupts a regulatory motif (R), whether the variant is located in the candidate gene (G) or the candidate gene is the nearest gene to the variant (N), and whether the candidate gene is supported by biological evidence in the literature (L) or by expression data obtained in this study (E). For all three regions where both trait and variant have appropriate equivalents in previous GWASs, our findings replicated with nominal significance ($p < 0.05$) or showed a trend in the same direction (see also Table S2). BETA, effect; Chr, chromosome; EA, effect allele; EAF, effect allele frequency; INFO, imputation quality, with 1 for directly genotyped variants; n, number of individuals with genotype and immune phenotype; NEA, non-effect allele; Pos, position in GRCh37; rsID, reference SNP identification; SE, standard error; % var, percentage of variance explained.

for the T cell transcription factor MEF2 (Blaeser et al., 2000) and was associated with Th2 frequency in humans (Figures 3A, 3D, and 3G).

SNP rs1457990, associated with Th17 frequency, is located in an intergenic region on chromosome 14 but is highly conserved as predicted by both genomic evolutionary rate profiling (GERP) and site-specific phylogenetic analysis (SiPhy) bioinformatics tools. We measured expression of candidate genes within a 1-Mb interval of the variant (*STON2*, *SEL1L*, and *LINC01467*) in Th17 and Th1 cells differentiated from naive $CD4^+$ T cells. We included *IL17A* and *IFNG* as positive controls for Th17 and Th1 cells, respectively. We subsequently demonstrated that *STON2* was the only chromosome 14 candidate gene differentially expressed in Th17 cells. Indeed, *STON2* was upregulated more than two-fold in Th17 versus Th1 cells (2.23 ± 0.19, $p = 0.0035$), suggesting a cell-intrinsic basis of variance in Th17 frequency (Figures 3B, 3E, and 3H).

Association with the frequency of $Foxp3^+$ Treg cells undergoing proliferation was seen for variant rs10853728. Of note, the minor allele was associated with an increase in percentage of proliferating Treg cells, but not overall Treg cell frequency (Figure 1B). This is a singleton variant, in weak LD ($r^2 < 0.2$) with any other SNP in the region, but was directly genotyped with the cluster plot passing visual inspection. This variant maps to the locus of the lambda interferons (IFN-λ), intergenic between interferon-lambda-2 (*IFNL2*) and interferon-lambda-4 (*IFNL4*) (Figures 3C and 3F).

LARP1B and *SP4* Are Involved in B Cell Differentiation

sjKREC excision circles allow quantification of newly formed B cells at the DNA level. Genome-wide significant association for sjKREC levels was seen for multiple variants throughout the *LARP1B* gene, with strongest association for the insertion or deletion variant rs373482106 (Figures 4A and 4C). Comparison with flow cytometry measurements showed sjKREC levels were correlated most strongly with B cells ($r^2 = 0.33$) and naive B cells ($r^2 = 0.22$), and nominal association in the same direction was observed for these cell types (Figure 1B). The region of association overlaps with an eQTL region for *LARP1B* in LCL (Grundberg et al., 2012). LARP1B belongs to the La-related protein family (LARP) with roles in transcription and/or translation. We demonstrated that treatments known to induce the formation of early B cells, such as interferon-beta (Dooley et al., 2016), increase sjKREC excision circles and decrease *LARP1B* gene expression levels, and we observed an inverse correlation between *LARP1B* gene expression and sjKREC levels (Figures 4E–4G).

Variant rs917812 associated with memory B cells maps adjacent to several genes, including *SP4*, *SP8*, and long-coding RNA *LINC01162* (Figures 4B and 4D). We measured gene expression *ex vivo* in four B cell subsets (transitional, naive, memory, and switched memory) isolated from healthy donors and found only *SP4* to be highly expressed in all B cell subsets (Figure 4H).

Genetic Control of Pro-inflammatory Cytokine Production

Our study also investigated genetic variants underlying baseline differences in *ex vivo* plasma cytokine levels in healthy individuals. SNP rs9324185 was associated at genome-wide significance with plasma interleukin-6 (IL-6) levels (Figures 5A and 5B). In line with the previously described phenotypic clustering of pro-inflammatory cytokines (Carr et al., 2016),

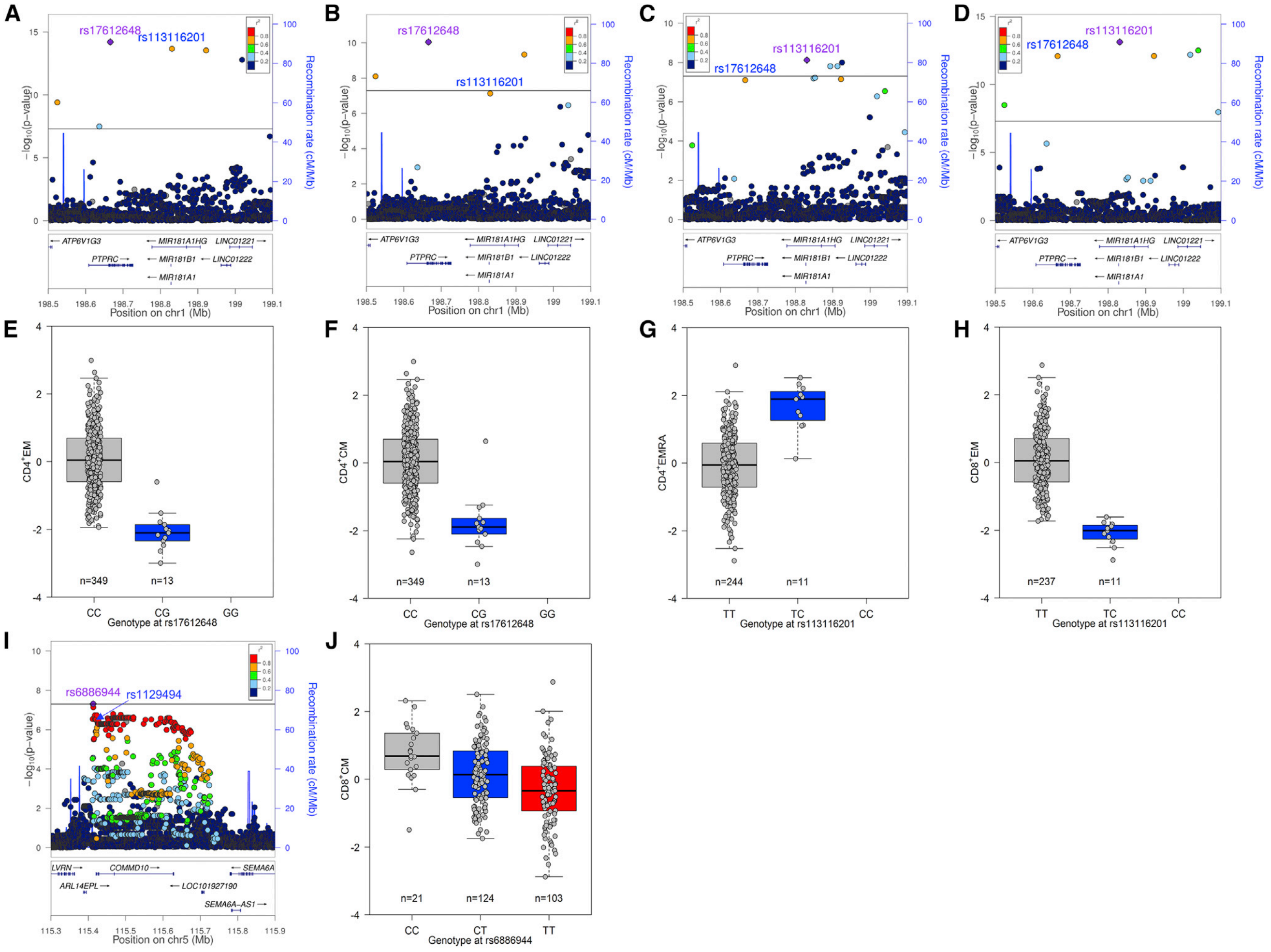

Figure 2. Coding and Splicing Variants Involved in T Cell Memory Differentiation

(A–H) Regional association plots (A–D) and boxplots (E–H) for *PTPRC* variants with $CD4^+$ effector memory (EM) T cells (A and E), $CD4^+$ central memory (CM) T cells (B and F), $CD4^+$ terminally differentiated (EMRA) T cells (C and G), and $CD8^+$ EM cells (D and H). Variant rs17612648 disrupts an exonic splicing silencer for *PTPRC* exon 4 (CD45RA splice form) and is in LD with rs113116201 (see also Table S3).

(I and J) Regional association plot (I) and boxplot (J) for the *COMMD10* region with $CD8^+$ CM cells. The lead variant rs6886944 is in high LD with synonymous coding variant rs1129494. In regional association plots, the x axis depicts the position on the chromosome and RefSeq genes, the left y axis indicates the negative logarithm of the p value for each variant (with the horizontal line corresponding to genome-wide significance or $p < 5 \times 10^{-8}$), and the right y axis shows recombination rates. The lead variant is indicated with a purple diamond and text, other variants of interest are indicated in blue text, and LD of other variants with the lead variant is color-coded based on r^2 in the 1000 Genomes November 2014 European (EUR) database. In boxplots, boxes indicate median and interquartile range, with whiskers extending to 1.5× the interquartile range.

the same variant was associated at nominal significance with other pro-inflammatory cytokines (TNF-α, IL-8, IL-2, and IFNG) (Figure 1B). No association (not even a trend) was seen for IL-6 gene expression levels in RNA extracted from peripheral blood mononuclear cells (PBMCs) (Figure 5C), suggesting a possible post-transcriptional mechanism or a non-hematopoietic source of IL-6 underlying the association with IL-6 protein levels. The variant is located within the promoter region of the most common *ADGRL2* splice form and within an intron of an alternatively transcribed splice form. The same locus has been associated with pediatric autoimmune diseases (Li et al., 2015), although correlation between the variant increasing IL-6 and the autoimmune disease risk variant is poor ($r^2 = 0.055$).

Genotype-Immune Phenotype Associations Shed Light on Mechanism of Action of Susceptibility Variants for Immune-Related Disorders

Beyond the associations reaching most robust genome-wide significance ($p < 5 \times 10^{-8}$), suggestive effects have been shown to explain an important proportion of variance in human traits (Shi et al., 2016; Yang et al., 2010) and to be enriched for true effects eventually reaching genome-wide significance with larger sample size or replication (International Multiple Sclerosis

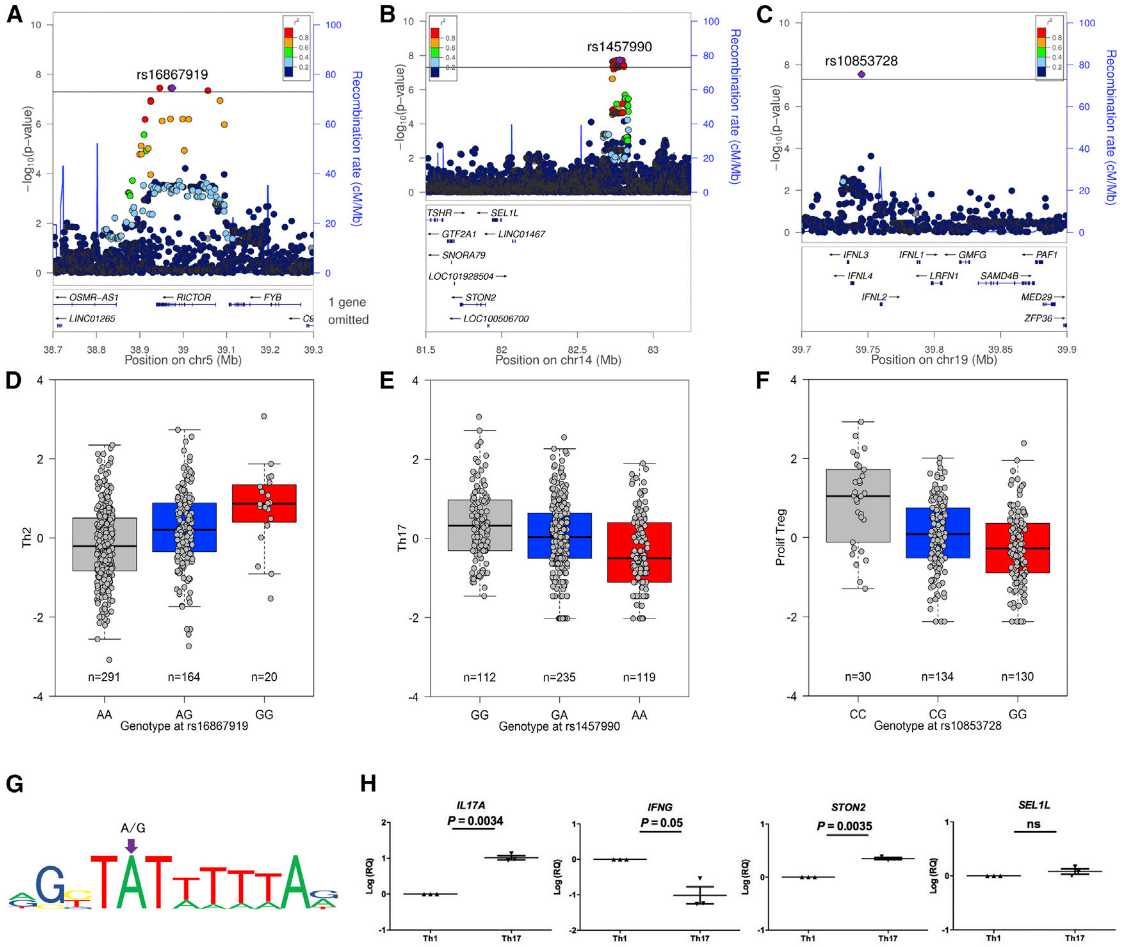

Figure 3. Genetic Variants Associated with T Helper Subset Differentiation and Proliferation

(A–G) Regional association (A–C) and boxplots (D–F) for T helper 2 (Th2) (A and D), T helper 17 (Th17) (B and E), and proliferating regulatory T cells (Tregs) (C and F). Legends as in Figure 2.

(G) Lead associated variant in *RICTOR* is predicted to disrupt the T cell transcription factor MEF2-binding site.

(H) Among three candidate genes (*STON2*, *SEL1L*, and *LINC01467*) within a 1-Mb interval in the chromosome 14 region, *STON2* was the only gene differentially expressed in Th17 versus Th1 cells differentiated from naive CD4$^+$ T cells. Expression of *LINC01467* was undetectable and not shown. *IL17A* and *IFNG* were included as positive controls for Th17 and Th1 cells, respectively. Mean and SEM for triplicate measurements from three donors are shown; relative quantity (RQ) was normalized using a T cell housekeeping gene (*RPL13A*) and was log-transformed for analysis.

Genetics Consortium (IMSGC) et al., 2013, 2017). Among 10,984 independent ($r^2 < 0.1$) autosomal lead signals associated with at least one phenotype, 39% fall within the category of less common variants (Table S4). GWAS have enabled extensive progress in the identification of risk variants for immune-related disorders, yet understanding their mechanism of action is a current key challenge. As known risk variants have a higher prior probability of association, we explored their overlap with suggestive variants. Specific genotype-immune phenotype associations and directions of effect thereby provide potential proximal inflammatory mediators between the *cis*-acting impact of the polymorphism and immune disease susceptibility (Tables 3 and S5).

Five variants were associated with levels of cytokines or cytokine-producing T cells. A well-established rheumatoid arthritis susceptibility variant near *CTLA4* (Gregersen et al., 2009) decreases CTLA4 levels in T cells (Kasela et al., 2017); here, we identify how this reduced immune checkpoint function is associated with increased levels of IL-2-producing CD8$^+$ T cells. Three variants increasing TNF-α correspond to risk variants for a range of autoimmune or putative autoimmune diseases (Baurecht et al., 2015; Dunstan et al., 2014; Freudenberg et al., 2011; Yamazaki et al., 2013; Zhang et al., 2015a). For SNP rs6930223, located in the *HBS1L-MYB* region and a known eQTL for *HBS1L*, the allele increasing GM-CSF-producing CD8$^+$ cells is protective against Hodgkin's lymphoma (Frampton et al., 2013).

The cluster of inflammatory bowel disease (IBD) risk genes that gave suggestive associations in our dataset appear to create a complex immune phenotype, with increased numbers of dendritic cells and a skewing away from the naive and memory subsets in CD8$^+$ T cells and B cells, respectively (Franke et al., 2010; Jostins et al., 2012; Liu et al., 2015). Moreover, the IBD

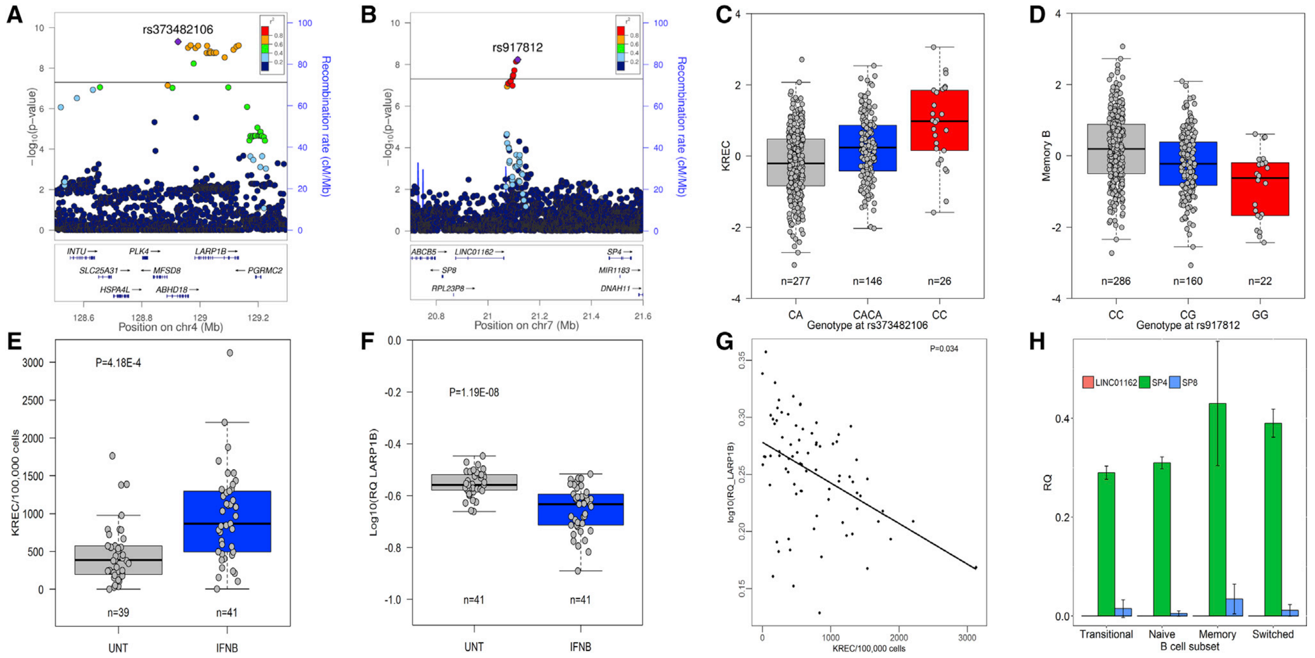

Figure 4. Genetic Variants Associated with B Cell Differentiation

(A–G) Regional association plots (A and B) and boxplots (C and D) for sjKREC levels (A and C) and memory B cells (B and D). Treatments known to increase early B cells such as interferon-beta (IFNB) compared to untreated multiple sclerosis patients (UNT) increased KREC levels (E) and decreased *LARP1B* gene expression (F), with an inverse correlation between *LARP1B* and KREC levels (G) (simplex measurements in PBMCs from 82 individuals).

(H) Among genes in the chromosome 7 region (*LINC01162*, *SP4*, and *SP8*), only *SP4* is highly expressed in B cell subsets. Mean and SD of gene expression levels (triplicate measurements from four donors) is depicted. Additional legend as in Figure 2.

risk locus *RTEL1-TNFRSF6B* has recently been fine-mapped to a variant disrupting the transcription factor binding site for EBF1 (Huang et al., 2017), involved in B cell differentiation (Györy et al., 2012), and we subsequently demonstrated the effect on B cell immune phenotype.

A cluster of B cell phenotype associations were shared with immune disorders of the barrier surfaces. A *CLEC16A* variant associated with increased levels of transitional B cells elevates the risk for atopic dermatitis, asthma, and hay fever (Paternoster et al., 2015), and a chromosome 15 locus leading to higher plasmablast levels is associated with airway obstruction in smokers (Lee et al., 2015; Lutz et al., 2015).

Suggestive associations in the human leukocyte antigen (HLA) region overlapping with disease loci (Tables 3 and S6) included the above-described variant for TNF-α levels and a risk variant for systemic sclerosis near HLA-DRB5 and HLA-DRB6 (Gorlova et al., 2011) associated with a higher percentage of lymphocytes. Finally, the HLA-DPA1*0103 allele associated with protection against hepatitis B (Kamatani et al., 2009) was correlated with higher levels of memory B cells, a cell type involved in vaccine response to hepatitis B in the mouse model (Kulkarni et al., 2016). In contrast to the predominant role of HLA in immune disease associations, however, there was a relative absence of classical HLA alleles among the eight genome-wide signals and the 13 suggestive signals for known autoimmune disease associations identified in this study.

DISCUSSION

In our GWAS of 54 immune traits in 489 healthy individuals, we identified eight genome-wide significant genotype-cellular immune phenotype associations that had not previously been reported. These associations were particularly enriched for functionally relevant adaptive immune phenotypes such as maturation and differentiation stages of B and T cells and Th2, Th17, and Treg cell subsets highly relevant in health and disease. For all eight associations, it was possible to identify the biologically most likely candidate gene through a combination of bioinformatics and functional analyses. The basis for understanding human immunology was until recently largely founded upon animal models. Intrinsic limitations in the fidelity of models to disease processes (i.e., interspecies barriers) have hampered translational potential, thereby spurring the call for characterization of the human immune system (Roep et al., 2012). Our study addresses this call, and two of our genome-wide significant associations provided the crucial first evidence translating key adaptive immune pathways with clinical implications from animal models to humans. Moreover, our associations identified recently described but still poorly characterized protein families as key immune regulators in humans. Signaling cascades implicated by these associations as critical in control of the adaptive immune system are the mTOR signaling complex 1 in B cells and the mTOR signaling complex 2 and nuclear factor κB (NF-κB) pathway in T cells.

OPEN ACCESS
CellPress

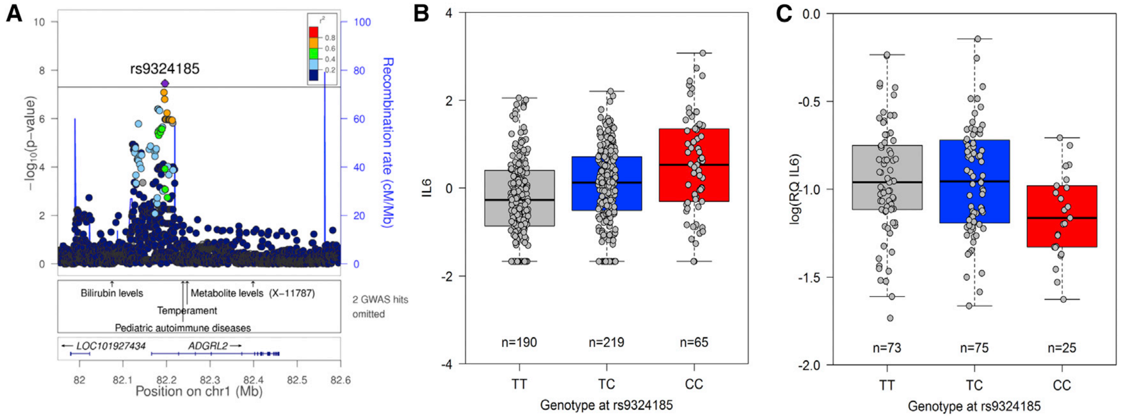

Figure 5. Genetic Control of Pro-inflammatory Cytokine Production

(A) Regional association plot for *ex vivo* plasma interleukin-6 levels additionally depicting known GWAS hits in this region, including a variant associated with pediatric autoimmune diseases but in weak LD ($r^2 = 0.055$).

(B and C) Boxplot for *ex vivo* plasma interleukin-6 levels (B) and interleukin-6 gene expression (C) in RNA extracted from PBMCs (simplex measurements from 173 individuals) ($p = 0.16$). Additional legend as in Figure 2.

We found a coding variant in *COMMD10* associated with CD8$^+$ T cell differentiation into memory cells. Members of the *COMMD* family interact with NF-κB (Burstein et al., 2005), a pathway recently shown to be essential for T cell memory (Knudson et al., 2017). CD4$^+$ T helper cells differentiate into functionally distinct subsets characterized by a unique cytokine expression pattern and a lineage-associated transcription factor network. We identified genetic variants underlying baseline variation between healthy individuals in Th2, Th17, and proliferating Treg cell subsets, subsets with crucial roles in the defense against infection and susceptibility to allergy and autoimmunity. We demonstrated association of an intergenic chromosome 14 variant with Th17 and implicated *STON2*, for which we showed significant upregulation in Th17 versus Th1 cells, in controlling this interindividual variation in baseline Th17 frequency. *In vitro*, *STON2*, a component of the endocytic machinery (Martina et al., 2001), is positively regulated by IRF4 and co-targeted by IRF4 and basic leucine zipper transcription factor ATF-like (BATF) when promoting Th17 differentiation through cooperation (Glasmacher et al., 2012). Our data hence extend the signaling cascade involving known Th17 controllers IRF4 and BATF in humans with *STON2* and demonstrate that this pathway is variant *in vivo* in humans. Deleting T cell mTORC2 signaling in T-*Rictor*$^{-/-}$ mice maintains their ability to differentiate into Th1 and Th17 cells but renders T cells unable to become Th2 cells (Delgoffe et al., 2011). This previous observation in mice is translated into humans in our study and provides a mechanistic explanation for the association observed here for a variant disrupting a MEF2 transcription binding site in the *RICTOR* gene with baseline Th2 frequency. Treatment of mouse immature dendritic cells with interferon-lambda *in vitro* instructs these cells toward a developmental program with the capacity to trigger the proliferation of Treg cells (Mennechet and Uzé, 2006). The genotype association between the interferon-lambda locus and the proliferation of Treg cells in healthy individuals described here indicates that in humans expression of lambda interferons is variant *in vivo* in a biologically relevant range capable of influencing immune suppression. Of note, interferon-lambda stimulates the proliferation of preexisting CD25$^+$Foxp3$^+$ T cells rather than driving their generation *de novo* (Mennechet and Uzé, 2006), as also reflected in our data, providing an attractive therapeutic intervention point.

On the basis of our genome-wide significant associations, still poorly characterized protein families emerge for their role in the control of B cell differentiation. First, we observed association of variants throughout *LARP1B* with newly formed B cells as measured by sjKREC excision circles. LARP1B is still poorly characterized but highly resembles LARP1 (Stavraka and Blagden, 2015). LARP1 acts downstream of mTORC1 (Fonseca et al., 2015; Hong et al., 2017), which, in addition to its well-known role in T cell metabolism and differentiation, controls early B cell development, survival, and metabolism in mice (Iwata et al., 2016). Our data now suggest an analogous function for the homolog LARP1B in early B cell development in humans. Our data on the effect of treatments known to induce early B cell development indicated an inverse correlation, with increased levels of LARP1B blocking early B cell differentiation. An intergenic variant on chromosome 7 was associated with memory B cell differentiation, and expression levels in B cells implicated the adjacent *SP4* gene, which is known to downregulate expression of the B cell differentiation factor *BCL2* (Hedrick et al., 2016; Nuñez et al., 1991).

Our study identified a genetic variant regulating baseline *ex vivo* IL-6 levels. Our observations are in line with a previous study on *in vitro* cytokine levels after stimulation, where IL-6 similarly showed the strongest interindividual variation and genetic influence, and nearly all cytokine QTLs were trans-QTLs altering cytokine production indirectly rather than *cis*-QTLs altering expression of the cytokine gene itself (Li et al., 2016). However, genetic determinants in stimulated conditions appear distinct to those in baseline conditions and involve pattern recognition and antigen processing pathways. *ADGRL2*, which is associated with baseline IL-6 protein levels, is also known as latrophilin-2 and belongs to a branch of adhesion G-protein-coupled

Table 3. Genotype-Immune Phenotype Correlations for Known Immune Disease Susceptibility Loci

Chr	Pos	rsID	EA	NEA	EAF	BETA (SE)	p value	Trait	Disease	Variants	Genes
2	204690355	rs231746	C	G	0.49	0.29 (0.06)	4.68×10^{-6}	$CD8^+$ $IL2^+$	RA (+)	rs231735*T	*CTLA4*
3	18752031	rs55845060	C	T	0.17	0.35 (0.08)	8.02×10^{-5}	TNF-α	Ps (+)	rs68080462*C	*SATB1*
6	32590924	rs3129763	A	G	0.26	0.35 (0.08)	2.93×10^{-5}	lymphocyte	SSc (+)	rs3129763	HLA class II
6	32680928	rs7765379	T	G	0.11	0.51 (0.10)	8.90×10^{-7}	TNF-α	RA, CD, EF (+)	rs7765379	HLA class II
6	33037085	HLA-DPA1*0103	P	A	0.80	0.32 (0.08)	6.73×10^{-5}	memory B	hepatitis B (−)	HLA-DPA1*0103	HLA-DPA1
6	135424203	rs6930223	G	T	0.54	−0.48 (0.12)	8.39×10^{-5}	$CD8^+$ GM-CSF^+	HL (+)	rs7745098*G	*HBS1L-MYB*
10	94502244	rs11187157	C	T	0.43	0.29 (0.07)	4.30×10^{-5}	mDCs	IBD (+)	rs11187157*C	*KIF11-HHEX*
10	94436851	rs7911264	T	C	0.53	0.31 (0.06)	3.98×10^{-6}	mDCs	IBD (+)	rs7911264*C	*KIF11-HHEX*
12	26691549	rs9668498	A	G	0.25	0.30 (0.08)	6.22×10^{-5}	TNF-α	KB (+)	rs10842750*A	*ITPR2*
15	78785944	rs373948468	ATT	AT	0.31	0.54 (0.12)	2.17×10^{-5}	plasmablast	COPD (+)	rs17484524*G, rs7181486*C	*IREB2*
16	11223454	rs11645657	C	G	0.45	0.59 (0.12)	4.89×10^{-6}	transitional B	AD, HF, asthma (+)	rs2041733*T	*CLEC16A*
16	50756881	rs2076756	G	A	0.32	−0.31 (0.08)	7.63×10^{-5}	$CD8^+$ naive	CD (+)	rs2076756*G	*NOD2*
20	62347191	rs62217799	T	G	0.69	−0.29 (0.07)	4.47×10^{-5}	memory B	CD (+)	rs4809330*G	*TNFRSF6B-RTEL1*

Suggestive ($p < 10^{-4}$) genotype-immune phenotype correlations in LD ($r^2 > 0.8$) with established immune disease susceptibility SNPs (+, susceptibility; −, protection). See also Tables S4 and S6 for all non-HLA and HLA suggestive associations, respectively, and Table S5 for all suggestive associations in LD with a variant for any disease in the EBI GWAS catalog. BETA, effect; EA, effect allele (P indicates present and A absent for HLA allele); EAF, effect allele frequency; NEA, non-effect allele; Pos, position in GRCh37; rsID, reference SNP identification; SE, standard error. Traits and disease associations: AD, atopic dermatitis (Paternoster et al., 2015), asthma (Paternoster et al., 2015); CD, Crohn disease (Franke et al., 2010; Yamazaki et al., 2013); COPD, chronic obstructive pulmonary disorder obstruction (Lee et al., 2015; Lutz et al., 2015); EF, enteric fever (Dunstan et al., 2014), hepatitis B (Kamatani et al., 2009); HF, hay fever (Paternoster et al., 2015); HL, Hodgkin lymphoma (Frampton et al., 2013); IBD, inflammatory bowel disease (Jostins et al., 2012; Liu et al., 2015); KB, Kashin-Beck disease (Zhang et al., 2015a); mDCs, myeloid dendritic cells; Ps, psoriasis (Baurecht et al., 2015); RA, rheumatoid arthritis (Freudenberg et al., 2011; Gregersen et al., 2009); SSc, systemic sclerosis (Gorlova et al., 2011).

receptors whose precise functions remain poorly characterized. Latrophilin-1 enhances IL-6 release through exocytosis in acute myeloid leukemia, but (importantly) not in healthy human leukocytes (Sumbayev et al., 2016). Our data now imply the homolog latrophilin-2 in regulating IL-6 secretion in baseline conditions in healthy human individuals. The absence of any association or trend for IL-6 PBMC gene expression levels suggests a post-transcriptional mechanism or a non-hematopoietic source.

Twin and family-based studies provide evidence for both genetic and environmental factors contributing to the human immune system (Brodin et al., 2015; Mangino et al., 2017; Orrù et al., 2013; Roederer et al., 2015). Four GWASs, with different ethnic composition, independent generation of immune phenotyping platforms, different definitions for immunological variables, and different inclusion of protein and cellular variables from the innate and/or adaptive immune system, have identified a total of 38 distinct loci associated with at least one immune trait (Aguirre-Gamboa et al., 2016; Orrù et al., 2013; Patin et al., 2018; Roederer et al., 2015), and our study adds eight additional loci with a particular focus on cell subsets relevant to immune disease. Studies observed associations with mainly protein QTLs (Patin et al., 2018), mainly cellular immune variables (Aguirre-Gamboa et al., 2016; Orrù et al., 2013) (our study) or both (Roederer et al., 2015), for predominantly the innate (Patin et al., 2018), adaptive (Aguirre-Gamboa et al., 2016; Orrù et al., 2013) (our study), or both immune systems (Roederer et al., 2015) and for common variants (Aguirre-Gamboa et al., 2016; Orrù et al., 2013; Patin et al., 2018; Roederer et al., 2015) or common and less common variants (our study).

GWASs have enabled extensive progress in the identification of risk variants for immune disorders, yet understanding the mechanism of action of these variants is a current key challenge, with indications on the putative disease mechanism known for only a small subset of established disease variants. Our results provide potential proximal inflammatory mediators between the *cis*-acting impact of a polymorphism and its established effect on immune disease susceptibility. These mechanisms implicate in particular checkpoints that are genetically variable in the physiologically relevant range and control cytokine levels and frequencies of cytokine-producing T cells. Variants controlling TNF-α levels are associated with a range of autoimmune diseases such as psoriasis, rheumatoid arthritis, and Crohn disease in which the role of TNF-α is known (Baurecht et al., 2015; Dunstan et al., 2014; Freudenberg et al., 2011; Yamazaki et al., 2013), and also with Kashin-Beck disease, corroborating its suspected inflammatory nature (Zhang et al., 2015a). Of note, we previously demonstrated that *ITPR2*, here associated with TNF-α, controls pro-inflammatory cytokine production in mice (Staats et al., 2016). The function of CTLA4 in suppressing IL-2 production has been long established (Krummel and Allison, 1996), but our

results demonstrate that this checkpoint is genetically variable in the physiologically relevant range of IL-2 suppression in humans, suggesting a mechanistic explanation for association of the *CTLA4* variant with rheumatoid arthritis. Natural genetic variation in GM-CSF-producing $CD8^+$ levels confers protection against Hodgkin lymphoma.

The relative absence of distinct HLA signals in our data despite known disease associations may be explained by the population, rather than clonal, level of immunophenotyping in our study. Our data suggest that overall, HLA variation does not change the global composition of the immune system. This is in line with other studies demonstrating an effect of HLA variation on the surface expression of HLA molecules in innate immune cells rather than on the frequencies of circulating immune cells (Patin et al., 2018). For exceptions such as an HLA SNP associated with rheumatoid arthritis, Crohn disease, and enteric fever (Dunstan et al., 2014; Freudenberg et al., 2011; Yamazaki et al., 2013), our observation of association with systemic TNF-α levels suggests a different mechanistic association instead of the classical hypothesis of modifying disease risk via antigen-specific effects.

The genotype-immune phenotype correlations in our study have important clinical implications. Genome-wide significant findings provide leads for therapeutic strategies and their translation to humans. The identification of phenotypic effects of these variants on key traits demonstrates that these genes are immunological fulcrum, where partial alterations drive physiological outcomes, an attractive property when assessing druggability. Examples are STON2 inhibition to lower Th17 levels critical in several autoimmune diseases, RICTOR stimulation to induce an anti-inflammatory and pro-regenerative context, or RICTOR inhibition in the case of Th2-mediated allergy or asthma, interferon-lambda provision to stimulate the proliferation of existing Treg cells (an attractive therapeutic intervention point for autoimmune disorders), blockade of early B cell differentiation by the La-related family homolog LARP1B, and blockade of exocytosis and release of pro-inflammatory cytokines by interfering with the latrophilin ADGRL2. Increased TNF-α production as the putative mechanism of action of rheumatoid arthritis and Crohn disease risk variants mirrors the success of anti-TNF therapies, whereas the higher levels of GM-CSF-producing $CD8^+$ T cells associated with Hodgkin lymphoma protection support recent GM-CSF treatment strategies (Schuster et al., 2008).

Whereas a dataset of 489 individuals with extensive immune characterization is considered exceptionally large from an immunological point of view, it is smaller compared to current-day disease GWASs. However, this is compensated by the effects of genetic variation on quantitative traits in the human immune system (median 10% of variance) being substantially larger than on disease susceptibility, in line with a previous study (Orrù et al., 2013). This has indeed enabled us to identify eight genome-wide significant genotype-immune phenotype associations. Mapping of genetic factors controlling variation in the immune system is still in the initial discovery phase, with replication across studies yet novel and strong associations emerging from each study (Liston and Goris, 2018). Our GWAS used a latest-generation imputation-based array designed to impute both common (>5%) and less common (1%–5%) variants. Our most significant association was indeed a splicing variant with MAFs of 2%, and 39% of suggestive lead variants were less common. As most previous GWASs have captured mainly common variants, it remains to be seen whether application of the same type of array and/or whole-genome sequencing to the study of disease susceptibility will implicate more of these less common variants affecting immune phenotype in the susceptibility to disease.

STAR★METHODS

Detailed methods are provided in the online version of this paper and include the following:

- KEY RESOURCES TABLE
- CONTACT FOR REAGENT AND RESOURCE SHARING
- EXPERIMENTAL MODEL AND SUBJECT DETAILS
  - Human subjects
- METHOD DETAILS
  - Flow cytometry phenotyping
  - Serological assessment
  - sjKREC and sjTREC levels
  - Immune phenotype data processing
  - Genotyping
  - Expression experiments
- QUANTIFICATION AND STATISTICAL ANALYSIS
  - Sample size and power
  - Genotyping quality control
  - Genotype imputation
  - GWAS analysis
  - Conditional analysis and LD pruning
  - Variant annotation
  - Expression analysis
- DATA AND SOFTWARE AVAILABILITY

SUPPLEMENTAL INFORMATION

Supplemental Information includes two figures and six tables and can be found with this article online at https://doi.org/10.1016/j.celrep.2018.09.048.

ACKNOWLEDGMENTS

We thank Prof. Carine Wouters and Prof. Isabelle Meyts for sample collection and the High-Throughput Genomics Group at the Wellcome Trust Centre for Human Genetics (funded by Wellcome Trust grant reference 090532/Z/09/Z) for the generation of the sequencing data. M.V. is a PhD Fellow, V.L. is a Research Associate, and B.D. and P.V.D. are Clinical Investigators of the Research Foundation Flanders (FWO-Vlaanderen). L.V.H. holds a PhD Fellowship of the Belgian Charcot Foundation. A.G. and B.D. are supported by the Research Council KU Leuven (C24/16/045) and the Research Foundation Flanders (G.0734.15). A.G. is supported by the Belgian Charcot Foundation, MS Liga Vlaanderen, and the Queen Elisabeth Medical Foundation (Research Grant and Prize Viscountess Valine de Spoelberch). A.G., B.D., and A.L. are supported by a Research Grant from Novartis for this project. A.L. is supported by the ERC. P.B. is supported by the National Institute for Health Research (NIHR) Oxford Biomedical Research Unit (BRC). The views expressed are those of the authors and not necessarily those of the NHS, NIHR, or Department of Health. The computational resources and services used in this work were provided by the VSC (Flemish Supercomputer Center), funded by the Research Foundation Flanders (FWO-Vlaanderen) and the Flemish Government (department EWI). A subset of the graphical abstract was obtained from http://www.somersault1824.com.

AUTHOR CONTRIBUTIONS

J.E.G.-P., L.C., K.M., T.P., S.H.-B., G.B., B.D., and J.D. obtained samples and data. V.L., I.S., L.V.H., M.V., K.H., M.M., and A.G. designed and performed statistical analyses. M.V. and S.H.-B. contributed to literature search and data interpretation. P.V.D., P.B., and B.D. provided resources and supervised parts of the study. A.G. wrote the first draft of the manuscript. A.L. and A.G. designed and supervised the study. All co-authors critically revised the manuscript.

DECLARATION OF INTERESTS

B.D. has received consulting fees and/or funding from Biogen Idec, Sanofi-Aventis, and Teva. B.D., A.G., and A.L. have received research funding from Novartis, and B.D. and A.G. have received travel or consulting fees and/or research funding from Merck and Roche. A.L.'s spouse is an ex-employee of UCB. The remaining authors declare no competing interests.

Received: June 13, 2018
Revised: August 16, 2018
Accepted: September 12, 2018
Published: October 16, 2018

REFERENCES

Abecasis, G.R., Auton, A., Brooks, L.D., DePristo, M.A., Durbin, R.M., Handsaker, R.E., Kang, H.M., Marth, G.T., and McVean, G.A.; 1000 Genomes Project Consortium (2012). An integrated map of genetic variation from 1,092 human genomes. Nature *491*, 56–65.

Aguirre-Gamboa, R., Joosten, I., Urbano, P.C.M., van der Molen, R.G., van Rijssen, E., van Cranenbroek, B., Oosting, M., Smeekens, S., Jaeger, M., Zorro, M., et al. (2016). Differential effects of environmental and genetic factors on T and B cell immune traits. Cell Rep. *17*, 2474–2487.

Baurecht, H., Hotze, M., Brand, S., Büning, C., Cormican, P., Corvin, A., Ellinghaus, D., Ellinghaus, E., Esparza-Gordillo, J., Fölster-Holst, R., et al.; Psoriasis Association Genetics Extension (2015). Genome-wide comparative analysis of atopic dermatitis and psoriasis gives insight into opposing genetic mechanisms. Am. J. Hum. Genet. *96*, 104–120.

Blaeser, F., Ho, N., Prywes, R., and Chatila, T.A. (2000). Ca(2+)-dependent gene expression mediated by MEF2 transcription factors. J. Biol. Chem. *275*, 197–209.

Boyle, A.P., Hong, E.L., Hariharan, M., Cheng, Y., Schaub, M.A., Kasowski, M., Karczewski, K.J., Park, J., Hitz, B.C., Weng, S., et al. (2012). Annotation of functional variation in personal genomes using RegulomeDB. Genome Res. *22*, 1790–1797.

Brodin, P., Jojic, V., Gao, T., Bhattacharya, S., Angel, C.J., Furman, D., Shen-Orr, S., Dekker, C.L., Swan, G.E., Butte, A.J., et al. (2015). Variation in the human immune system is largely driven by non-heritable influences. Cell *160*, 37–47.

Burstein, E., Hoberg, J.E., Wilkinson, A.S., Rumble, J.M., Csomos, R.A., Komarck, C.M., Maine, G.N., Wilkinson, J.C., Mayo, M.W., and Duckett, C.S. (2005). COMMD proteins, a novel family of structural and functional homologs of MURR1. J. Biol. Chem. *280*, 22222–22232.

Carr, E.J., Dooley, J., Garcia-Perez, J.E., Lagou, V., Lee, J.C., Wouters, C., Meyts, I., Goris, A., Boeckxstaens, G., Linterman, M.A., and Liston, A. (2016). The cellular composition of the human immune system is shaped by age and cohabitation. Nat. Immunol. *17*, 461–468.

Delaneau, O., and Marchini, J.1000 Genomes Project Consortium; 1000 Genomes Project Consortium (2014). Integrating sequence and array data to create an improved 1000 Genomes Project haplotype reference panel. Nat. Commun. *5*, 3934.

Delgoffe, G.M., Pollizzi, K.N., Waickman, A.T., Heikamp, E., Meyers, D.J., Horton, M.R., Xiao, B., Worley, P.F., and Powell, J.D. (2011). The kinase mTOR regulates the differentiation of helper T cells through the selective activation of signaling by mTORC1 and mTORC2. Nat. Immunol. *12*, 295–303.

Dooley, J., Pauwels, I., Franckaert, D., Smets, I., Garcia-Perez, J.E., Hilven, K., Danso-Abeam, D., Terbeek, J., Nguyen, A.T.L., De Muynck, L., et al. (2016). Immunologic profiles of multiple sclerosis treatments reveal shared early B cell alterations. Neurol. Neuroimmunol. Neuroinflamm. *3*, e240.

Dunstan, S.J., Hue, N.T., Han, B., Li, Z., Tram, T.T., Sim, K.S., Parry, C.M., Chinh, N.T., Vinh, H., Lan, N.P., et al. (2014). Variation at HLA-DRB1 is associated with resistance to enteric fever. Nat. Genet. *46*, 1333–1336.

Euesden, J., Lewis, C.M., and O'Reilly, P.F. (2015). PRSice: Polygenic Risk Score software. Bioinformatics *31*, 1466–1468.

Fonseca, B.D., Zakaria, C., Jia, J.J., Graber, T.E., Svitkin, Y., Tahmasebi, S., Healy, D., Hoang, H.D., Jensen, J.M., Diao, I.T., et al. (2015). La-related protein 1 (LARP1) represses terminal oligopyrimidine (TOP) mRNA translation downstream of mTOR complex 1 (mTORC1). J. Biol. Chem. *290*, 15996–16020.

Frampton, M., da Silva Filho, M.I., Broderick, P., Thomsen, H., Försti, A., Vijayakrishnan, J., Cooke, R., Enciso-Mora, V., Hoffmann, P., Nöthen, M.M., et al. (2013). Variation at 3p24.1 and 6q23.3 influences the risk of Hodgkin's lymphoma. Nat. Commun. *4*, 2549.

Franke, A., McGovern, D.P., Barrett, J.C., Wang, K., Radford-Smith, G.L., Ahmad, T., Lees, C.W., Balschun, T., Lee, J., Roberts, R., et al. (2010). Genome-wide meta-analysis increases to 71 the number of confirmed Crohn's disease susceptibility loci. Nat. Genet. *42*, 1118–1125.

Freudenberg, J., Lee, H.S., Han, B.G., Shin, H.D., Kang, Y.M., Sung, Y.K., Shim, S.C., Choi, C.B., Lee, A.T., Gregersen, P.K., and Bae, S.C. (2011). Genome-wide association study of rheumatoid arthritis in Koreans: population-specific loci as well as overlap with European susceptibility loci. Arthritis Rheum. *63*, 884–893.

Glasmacher, E., Agrawal, S., Chang, A.B., Murphy, T.L., Zeng, W., Vander Lugt, B., Khan, A.A., Ciofani, M., Spooner, C.J., Rutz, S., et al. (2012). A genomic regulatory element that directs assembly and function of immune-specific AP-1-IRF complexes. Science *338*, 975–980.

Gorlova, O., Martin, J.E., Rueda, B., Koeleman, B.P., Ying, J., Teruel, M., Diaz-Gallo, L.M., Broen, J.C., Vonk, M.C., Simeon, C.P., et al.; Spanish Scleroderma Group (2011). Identification of novel genetic markers associated with clinical phenotypes of systemic sclerosis through a genome-wide association strategy. PLoS Genet. *7*, e1002178.

Gregersen, P.K., Amos, C.I., Lee, A.T., Lu, Y., Remmers, E.F., Kastner, D.L., Seldin, M.F., Criswell, L.A., Plenge, R.M., Holers, V.M., et al. (2009). REL, encoding a member of the NF-kappaB family of transcription factors, is a newly defined risk locus for rheumatoid arthritis. Nat. Genet. *41*, 820–823.

Grundberg, E., Small, K.S., Hedman, A.K., Nica, A.C., Buil, A., Keildson, S., Bell, J.T., Yang, T.P., Meduri, E., Barrett, A., et al.; Multiple Tissue Human Expression Resource (MuTHER) Consortium (2012). Mapping cis- and trans-regulatory effects across multiple tissues in twins. Nat. Genet. *44*, 1084–1089.

Györy, I., Boller, S., Nechanitzky, R., Mandel, E., Pott, S., Liu, E., and Grosschedl, R. (2012). Transcription factor Ebf1 regulates differentiation stage-specific signaling, proliferation, and survival of B cells. Genes Dev. *26*, 668–682.

Hedrick, E., Cheng, Y., Jin, U.H., Kim, K., and Safe, S. (2016). Specificity protein (Sp) transcription factors Sp1, Sp3 and Sp4 are non-oncogene addiction genes in cancer cells. Oncotarget *7*, 22245–22256.

Hong, S., Freeberg, M.A., Han, T., Kamath, A., Yao, Y., Fukuda, T., Suzuki, T., Kim, J.K., and Inoki, K. (2017). LARP1 functions as a molecular switch for mTORC1-mediated translation of an essential class of mRNAs. eLife *6*, e25237.

Howie, B.N., Donnelly, P., and Marchini, J. (2009). A flexible and accurate genotype imputation method for the next generation of genome-wide association studies. PLoS Genet. *5*, e1000529.

Huang, H., Fang, M., Jostins, L., Umićević Mirkov, M., Boucher, G., Anderson, C.A., Andersen, V., Cleynen, I., Cortes, A., Crins, F., et al.; International Inflammatory Bowel Disease Genetics Consortium (2017). Fine-mapping inflammatory bowel disease loci to single-variant resolution. Nature *547*, 173–178.

International Multiple Sclerosis Genetics Consortium (IMSGC) (2013). Analysis of immune-related loci identifies 48 new susceptibility variants for multiple sclerosis. Nat. Genet. *45*, 1353–1360.

International Multiple Sclerosis Genetics Consortium (IMSGC) (2017). The Multiple Sclerosis Genomic Map: role of peripheral immune cells and resident microglia in susceptibility. bioRxiv. https://doi.org/10.1101/143933.

Iwata, T.N., Ramírez, J.A., Tsang, M., Park, H., Margineantu, D.H., Hockenbery, D.M., and Iritani, B.M. (2016). Conditional disruption of raptor reveals an essential role for mTORC1 in B cell development, survival, and metabolism. J. Immunol. *197*, 2250–2260.

Jacobsen, M., Schweer, D., Ziegler, A., Gaber, R., Schock, S., Schwinzer, R., Wonigeit, K., Lindert, R.B., Kantarci, O., Schaefer-Klein, J., et al. (2000). A point mutation in PTPRC is associated with the development of multiple sclerosis. Nat. Genet. *26*, 495–499.

Jia, X., Han, B., Onengut-Gumuscu, S., Chen, W.M., Concannon, P.J., Rich, S.S., Raychaudhuri, S., and de Bakker, P.I. (2013). Imputing amino acid polymorphisms in human leukocyte antigens. PLoS ONE *8*, e64683.

Jostins, L., Ripke, S., Weersma, R.K., Duerr, R.H., McGovern, D.P., Hui, K.Y., Lee, J.C., Schumm, L.P., Sharma, Y., Anderson, C.A., et al.; International IBD Genetics Consortium (IIBDGC) (2012). Host-microbe interactions have shaped the genetic architecture of inflammatory bowel disease. Nature *491*, 119–124.

Kamatani, Y., Wattanapokayakit, S., Ochi, H., Kawaguchi, T., Takahashi, A., Hosono, N., Kubo, M., Tsunoda, T., Kamatani, N., Kumada, H., et al. (2009). A genome-wide association study identifies variants in the HLA-DP locus associated with chronic hepatitis B in Asians. Nat. Genet. *41*, 591–595.

Kasela, S., Kisand, K., Tserel, L., Kaleviste, E., Remm, A., Fischer, K., Esko, T., Westra, H.J., Fairfax, B.P., Makino, S., et al. (2017). Pathogenic implications for autoimmune mechanisms derived by comparative eQTL analysis of CD4+ versus CD8+ T cells. PLoS Genet. *13*, e1006643.

Knudson, K.M., Pritzl, C.J., Saxena, V., Altman, A., Daniels, M.A., and Teixeiro, E. (2017). NFκB-Pim-1-Eomesodermin axis is critical for maintaining CD8 T-cell memory quality. Proc. Natl. Acad. Sci. USA *114*, E1659–E1667.

Krummel, M.F., and Allison, J.P. (1996). CTLA-4 engagement inhibits IL-2 accumulation and cell cycle progression upon activation of resting T cells. J. Exp. Med. *183*, 2533–2540.

Kulkarni, S.P., Thanapati, S., Arankalle, V.A., and Tripathy, A.S. (2016). Specific memory B cell response and participation of $CD4^+$ central and effector memory T cells in mice immunized with liposome encapsulated recombinant NE protein based Hepatitis E vaccine candidate. Vaccine *34*, 5895–5902.

Lappalainen, T., Sammeth, M., Friedländer, M.R., 't Hoen, P.A., Monlong, J., Rivas, M.A., Gonzàlez-Porta, M., Kurbatova, N., Griebel, T., Ferreira, P.G., et al.; Geuvadis Consortium (2013). Transcriptome and genome sequencing uncovers functional variation in humans. Nature *501*, 506–511.

Lee, J.H., Cho, M.H., Hersh, C.P., McDonald, M.L., Wells, J.M., Dransfield, M.T., Bowler, R.P., Lynch, D.A., Lomas, D.A., Crapo, J.D., and Silverman, E.K.; COPDGene and ECLIPSE Investigators (2015). IREB2 and GALC are associated with pulmonary artery enlargement in chronic obstructive pulmonary disease. Am. J. Respir. Cell Mol. Biol. *52*, 365–376.

Li, Y.R., Li, J., Zhao, S.D., Bradfield, J.P., Mentch, F.D., Maggadottir, S.M., Hou, C., Abrams, D.J., Chang, D., Gao, F., et al. (2015). Meta-analysis of shared genetic architecture across ten pediatric autoimmune diseases. Nat. Med. *21*, 1018–1027.

Li, Y., Oosting, M., Smeekens, S.P., Jaeger, M., Aguirre-Gamboa, R., Le, K.T.T., Deelen, P., Ricaño-Ponce, I., Schoffelen, T., Jansen, A.F.M., et al. (2016). A functional genomics approach to understand variation in cytokine production in humans. Cell *167*, 1099–1110.e14.

Liang, L., Morar, N., Dixon, A.L., Lathrop, G.M., Abecasis, G.R., Moffatt, M.F., and Cookson, W.O. (2013). A cross-platform analysis of 14,177 expression quantitative trait loci derived from lymphoblastoid cell lines. Genome Res. *23*, 716–726.

Liston, A., and Goris, A. (2018). The origins of diversity in human immunity. Nat. Immunol. *19*, 209–210.

Liston, A., Carr, E.J., and Linterman, M.A. (2016). Shaping variation in the human immune system. Trends Immunol. *37*, 637–646.

Liu, J.Z., van Sommeren, S., Huang, H., Ng, S.C., Alberts, R., Takahashi, A., Ripke, S., Lee, J.C., Jostins, L., Shah, T., et al.; International Multiple Sclerosis Genetics Consortium (IMSGC); International IBD Genetics Consortium (2015). Association analyses identify 38 susceptibility loci for inflammatory bowel disease and highlight shared genetic risk across populations. Nat. Genet. *47*, 979–986.

Lotta, L.A., Gulati, P., Day, F.R., Payne, F., Ongen, H., van de Bunt, M., Gaulton, K.J., Eicher, J.D., Sharp, S.J., Luan, J., et al.; EPIC-InterAct Consortium; Cambridge FPLD1 Consortium (2017). Integrative genomic analysis implicates limited peripheral adipose storage capacity in the pathogenesis of human insulin resistance. Nat. Genet. *49*, 17–26.

Lutz, S.M., Cho, M.H., Young, K., Hersh, C.P., Castaldi, P.J., McDonald, M.L., Regan, E., Mattheisen, M., DeMeo, D.L., Parker, M., et al.; ECLIPSE Investigators; COPDGene Investigators (2015). A genome-wide association study identifies risk loci for spirometric measures among smokers of European and African ancestry. BMC Genet. *16*, 138.

Lynch, K.W., and Weiss, A. (2001). A CD45 polymorphism associated with multiple sclerosis disrupts an exonic splicing silencer. J. Biol. Chem. *276*, 24341–24347.

Mangino, M., Roederer, M., Beddall, M.H., Nestle, F.O., and Spector, T.D. (2017). Innate and adaptive immune traits are differentially affected by genetic and environmental factors. Nat. Commun. *8*, 13850.

Marchini, J., Howie, B., Myers, S., McVean, G., and Donnelly, P. (2007). A new multipoint method for genome-wide association studies by imputation of genotypes. Nat. Genet. *39*, 906–913.

Martina, J.A., Bonangelino, C.J., Aguilar, R.C., and Bonifacino, J.S. (2001). Stonin 2: an adaptor-like protein that interacts with components of the endocytic machinery. J. Cell Biol. *153*, 1111–1120.

Mennechet, F.J., and Uzé, G. (2006). Interferon-lambda-treated dendritic cells specifically induce proliferation of FOXP3-expressing suppressor T cells. Blood *107*, 4417–4423.

Nuñez, G., Hockenbery, D., McDonnell, T.J., Sorensen, C.M., and Korsmeyer, S.J. (1991). Bcl-2 maintains B cell memory. Nature *353*, 71–73.

O'Connell, J., Gurdasani, D., Delaneau, O., Pirastu, N., Ulivi, S., Cocca, M., Traglia, M., Huang, J., Huffman, J.E., Rudan, I., et al. (2014). A general approach for haplotype phasing across the full spectrum of relatedness. PLoS Genet. *10*, e1004234.

Orrù, V., Steri, M., Sole, G., Sidore, C., Virdis, F., Dei, M., Lai, S., Zoledziewska, M., Busonero, F., Mulas, A., et al. (2013). Genetic variants regulating immune cell levels in health and disease. Cell *155*, 242–256.

Paila, U., Chapman, B.A., Kirchner, R., and Quinlan, A.R. (2013). GEMINI: integrative exploration of genetic variation and genome annotations. PLoS Comput. Biol. *9*, e1003153.

Paternoster, L., Standl, M., Waage, J., Baurecht, H., Hotze, M., Strachan, D.P., Curtin, J.A., Bønnelykke, K., Tian, C., Takahashi, A., et al.; Australian Asthma Genetics Consortium (AAGC) (2015). Multi-ancestry genome-wide association study of 21,000 cases and 95,000 controls identifies new risk loci for atopic dermatitis. Nat. Genet. *47*, 1449–1456.

Patin, E., Hasan, M., Bergstedt, J., Rouilly, V., Libri, V., Urrutia, A., Alanio, C., Scepanovic, P., Hammer, C., Jönsson, F., et al.; Milieu Intérieur Consortium (2018). Natural variation in the parameters of innate immune cells is preferentially driven by genetic factors. Nat. Immunol. *19*, 302–314.

Price, A.L., Weale, M.E., Patterson, N., Myers, S.R., Need, A.C., Shianna, K.V., Ge, D., Rotter, J.I., Torres, E., Taylor, K.D., et al. (2008). Long-range LD can confound genome scans in admixed populations. Am. J. Hum. Genet. *83*, 132–135, author reply 135–139.

Pruim, R.J., Welch, R.P., Sanna, S., Teslovich, T.M., Chines, P.S., Gliedt, T.P., Boehnke, M., Abecasis, G.R., and Willer, C.J. (2010). LocusZoom: regional visualization of genome-wide association scan results. Bioinformatics *26*, 2336–2337.

Purcell, S., Neale, B., Todd-Brown, K., Thomas, L., Ferreira, M.A., Bender, D., Maller, J., Sklar, P., de Bakker, P.I., Daly, M.J., and Sham, P.C. (2007). PLINK: a tool set for whole-genome association and population-based linkage analyses. Am. J. Hum. Genet. *81*, 559–575.

R Development Core Team (2015). R: A Language and Environment for Statistical Computing (R Foundation for Statistical Computing).

Roederer, M., Quaye, L., Mangino, M., Beddall, M.H., Mahnke, Y., Chattopadhyay, P., Tosi, I., Napolitano, L., Terranova Barberio, M., Menni, C., et al. (2015). The genetic architecture of the human immune system: a bioresource for autoimmunity and disease pathogenesis. Cell *161*, 387–403.

Roep, B.O., Buckner, J., Sawcer, S., Toes, R., and Zipp, F. (2012). The problems and promises of research into human immunology and autoimmune disease. Nat. Med. *18*, 48–53.

Schuster, S.J., Venugopal, P., Kern, J.C., and McLaughlin, P. (2008). GM-CSF plus rituximab immunotherapy: translation of biologic mechanisms into therapy for indolent B-cell lymphomas. Leuk. Lymphoma *49*, 1681–1692.

Schwinzer, R., and Wonigeit, K. (1990). Genetically determined lack of CD45R-T cells in healthy individuals. Evidence for a regulatory polymorphism of CD45R antigen expression. J. Exp. Med. *171*, 1803–1808.

Shen-Orr, S.S., Furman, D., Kidd, B.A., Hadad, F., Lovelace, P., Huang, Y.W., Rosenberg-Hasson, Y., Mackey, S., Grisar, F.A., Pickman, Y., et al. (2016). Defective signaling in the JAK-STAT pathway tracks with chronic inflammation and cardiovascular risk in aging humans. Cell Syst. *3*, 374–384.e4.

Shi, H., Kichaev, G., and Pasaniuc, B. (2016). Contrasting the genetic architecture of 30 complex traits from summary association data. Am. J. Hum. Genet. *99*, 139–153.

Staats, K.A., Humblet-Baron, S., Bento-Abreu, A., Scheveneels, W., Nikolaou, A., Deckers, K., Lemmens, R., Goris, A., Van Ginderachter, J.A., Van Damme, P., et al. (2016). Genetic ablation of IP3 receptor 2 increases cytokines and decreases survival of SOD1G93A mice. Hum. Mol. Genet. *25*, 3491–3499.

Stavraka, C., and Blagden, S. (2015). The La-related proteins, a family with connections to cancer. Biomolecules *5*, 2701–2722.

Sudmant, P.H., Rausch, T., Gardner, E.J., Handsaker, R.E., Abyzov, A., Huddleston, J., Zhang, Y., Ye, K., Jun, G., Fritz, M.H., et al.; 1000 Genomes Project Consortium (2015). An integrated map of structural variation in 2,504 human genomes. Nature *526*, 75–81.

Sumbayev, V.V., Gonçalves Silva, I., Blackburn, J., Gibbs, B.F., Yasinska, I.M., Garrett, M.D., Tonevitsky, A.G., and Ushkaryov, Y.A. (2016). Expression of functional neuronal receptor latrophilin 1 in human acute myeloid leukaemia cells. Oncotarget *7*, 45575–45583.

Tsang, J.S., Schwartzberg, P.L., Kotliarov, Y., Biancotto, A., Xie, Z., Germain, R.N., Wang, E., Olnes, M.J., Narayanan, M., Golding, H., et al.; Baylor HIPC Center; CHI Consortium (2014). Global analyses of human immune variation reveal baseline predictors of postvaccination responses. Cell *157*, 499–513.

van Zelm, M.C., van der Burg, M., Langerak, A.W., and van Dongen, J.J. (2011). PID comes full circle: applications of V(D)J recombination excision circles in research, diagnostics and newborn screening of primary immunodeficiency disorders. Front. Immunol. *2*, 12.

Ward, L.D., and Kellis, M. (2012). HaploReg: a resource for exploring chromatin states, conservation, and regulatory motif alterations within sets of genetically linked variants. Nucleic Acids Res. *40*, D930–D934.

Weale, M.E. (2010). Quality control for genome-wide association studies. Methods Mol. Biol. *628*, 341–372.

Yamazaki, K., Umeno, J., Takahashi, A., Hirano, A., Johnson, T.A., Kumasaka, N., Morizono, T., Hosono, N., Kawaguchi, T., Takazoe, M., et al. (2013). A genome-wide association study identifies 2 susceptibility Loci for Crohn's disease in a Japanese population. Gastroenterology *144*, 781–788.

Yang, J., Benyamin, B., McEvoy, B.P., Gordon, S., Henders, A.K., Nyholt, D.R., Madden, P.A., Heath, A.C., Martin, N.G., Montgomery, G.W., et al. (2010). Common SNPs explain a large proportion of the heritability for human height. Nat. Genet. *42*, 565–569.

Yu, C.H., Pal, L.R., and Moult, J. (2016). Consensus genome-wide expression quantitative trait loci and their relationship with human complex trait disease. OMICS *20*, 400–414.

Zeller, T., Wild, P., Szymczak, S., Rotival, M., Schillert, A., Castagne, R., Maouche, S., Germain, M., Lackner, K., Rossmann, H., et al. (2010). Genetics and beyond—the transcriptome of human monocytes and disease susceptibility. PLoS ONE *5*, e10693.

Zhang, F., Wen, Y., Guo, X., Zhang, Y., Wang, X., Yang, T., Shen, H., Chen, X., Tian, Q., and Deng, H.W. (2015a). Genome-wide association study identifies ITPR2 as a susceptibility gene for Kashin-Beck disease in Han Chinese. Arthritis Rheumatol. *67*, 176–181.

Zhang, X., Joehanes, R., Chen, B.H., Huan, T., Ying, S., Munson, P.J., Johnson, A.D., Levy, D., and O'Donnell, C.J. (2015b). Identification of common genetic variants controlling transcript isoform variation in human whole blood. Nat. Genet. *47*, 345–352.

STAR★METHODS

KEY RESOURCES TABLE

REAGENT or RESOURCE	SOURCE	IDENTIFIER
Antibodies		
CCR7 G043H7	Biolegend	Cat# 353205; RRID: AB_10918624
CD11c 3.9	eBioscience	Cat# 46-0116-41; RRID: AB_10598361
CD123 6H6	eBioscience	Cat# 25-1239-41; RRID: AB_1257137
CD14 61D3	eBioscience	Cat# 48-0149-41; RRID: AB_1272120
CD24 ML5	Biolegend	Cat# 311121; RRID: AB_10915556
CD27 O323	eBioscience	Cat# 56-0279-41; RRID: AB_11149315
CD3 SK7	eBioscience	Cat# 56-0037-42; RRID: AB_10714978
CD31 WM-59	eBioscience	Cat# 12-0319-41; RRID: AB_10670623
CD38 HIT2	eBioscience	Cat# 17-0389-41; RRID: AB_1834354
CD4 RPA-T4	eBioscience	Cat# 61-0049-41; RRID: AB_2574521
CD8α RPA-T8	eBioscience	Cat# 56-0088-41; RRID: AB_11218867
CD19 HIB19	Biolegend	Cat# 302241; RRID: AB_2561381
CD45Ra HI100	eBioscience	Cat# 47-0458-41; RRID: AB_10853513
CD56 5.1H11	Biolegend	Cat# 362503; RRID: AB_2563912
CXCR5 J252D	Biolegend	Cat# 356903; RRID: AB_2561812
FOXP3 206D	Biolegend	Cat# 320113: RRID: AB_439753
γδTCR B1.1	eBioscience	Cat# 11-9959-41; RRID: AB_10669048
HLA-DR LN3	eBioscience	Cat# 47-9956-41; RRID: AB_1963604
IFNγ 4S.B3	eBioscience	Cat# 47-7319-41; RRID: AB_10853010
IgE IgE21	eBioscience	Cat# 11-6986-41; RRID: AB_10717661
IgM MHM-88	Biolegend	Cat# 314511; RRID: AB_961367
IL-17 eBio64DEC17	eBioscience	Cat# 11-7179-41; RRID: AB_10854885
IL-2 MQ1-17H12	eBioscience	Cat# 46-7029-41; RRID: AB_1834420
IL-4 8D4-8	eBioscience	Cat# 12-7049-41; RRID: AB_1548823
Ki67 B56	BD	Cat# 556027; RRID: AB_2266296
CD127 eBioRDR5	eBioscience	Cat# 13-1278-82; RRID: AB_657595
GM-CSF BVD-21C11	eBioscience	Cat# 502309; RRID: AB_11148950
IL-21 eBio3A3-N2	Biolegend	Cat# 12-7219-41; RRID: AB_1582261
Vα24Jα18 6B11	eBioscience	Cat# 12-5806-41; RRID: AB_1724174
Naive CD4+ T Cell Isolation Kit II, human	Miltenyi Biotec	130-094-131
T Cell Activation/Expansion Kit, human	Miltenyi Biotec	130-091-441
CD24 ML5	BioLegend	Cat# 311103; RRID: AB_314852
IgM SA-DA4	eBioscience	Cat# 12-9998-42; RRID: AB_11150964
CD14 TuK4	eBioscience	Cat# MHCD1418; RRID: AB_10371748
IgD IA6-2	BioLegend	Cat# 348209; RRID: AB_10683460
CD38 HIT2	eBioscience	Cat# 17-0389-42; RRID: AB_1834353
CD27 O323	eBioscience	Cat# 56-0279-42: RRID: AB_11044789
CD19 HIB19	BioLegend	Cat# 302209; RRID: AB_314239
Biological Samples		
Serum and PBMCs of healthy volunteers	Carr et al., 2016	N/A
DNA samples of healthy volunteers	This paper	N/A
PBMCs, DNA and RNA samples of healthy volunteers	This paper	N/A
DNA and RNA samples of multiple sclerosis patients	This paper	N/A

(*Continued on next page*)

Continued

REAGENT or RESOURCE	SOURCE	IDENTIFIER
Chemicals, Peptides, and Recombinant Proteins		
Lymphocyte separation medium	LSM, MP Biomedicals	0850494
Lymphoprep	StemCell Technologies	07861
10% DMSO	Sigma	D2650-100ML
Histopaque-1077	Sigma	10771
Fixation-permeabilization buffer	eBioscience	00-5523-00
PMA	Sigma	P8139
Ionomycin	Sigma	I3909-1ML
GolgiStop	BD Biosciences	554724
Cytofix/Cytoperm	BD Biosciences	55471
IL-2	Peprotech	200-02
IL-12	R&D Systems	219-IL-005
IL-1β	Peprotech	200-01B
IL-6	Peprotech	200-06
IL-23	Peprotech	200-23
EcoRI	New England Biolabs	R0101S
Trizol	Thermo Fisher	15596026
High-Capacity cDNA Reverse Transcription Kit	Thermo Fisher	4374967
Critical Commercial Assays		
BAFF Quantikine ELISA kit	R&D Systems	DBLYS0B
V-Plex human Proinflammatory panel MSD	Meso Scale Discovery	K15049D-2
Infinium HTS assay on Global Screening Array bead-chips	Illumina	https://www.illumina.com
Deposited Data		
Immune phenotypes of healthy volunteers	Carr et al., 2016	Raw Data Resource
Strand file for Illumina GSA array (“GSAMD-24v1-0_20011747_A1” file)	Developer: Will Rayner	http://www.well.ox.ac.uk/~wrayner/strand/
1000 Genomes reference datasets [Phase1 (2012); Phase 3 (2014)]	1000 Genomes Project Consortium, 2012; Sudmant et al., 2015	http://www.internationalgenome.org/
T1DGC reference panel for the HLA region, build 37	Jia et al., 2013	https://repository.niddk.nih.gov/studies/t1dgc-special/
ExSNP integrated eQTL database (“All processed eQTL data for each study” file)	Yu et al., 2016	http://www.exsnp.org/
Published dataset for splicing QTLs in whole blood (“ng.3220-S2.xlsx” file)	Zhang et al., 2015b	N/A
Oligonucleotides		
DNA detection assays for sjKREC	Thermo Fisher	Custom made
DNA detection assays for sjKREC	Thermo Fisher	Custom made
TaqMan Copy Number Reference Assay for human RNase P	Thermo Fisher	4403326
STON2 Gene Expression Assay	Thermo Fisher	Hs00263833_m1
SEL1L Gene Expression Assay	Thermo Fisher	Hs01071406_m1
LINC01467 Gene Expression Assay	Thermo Fisher	Hs04403614_m1
RPL13A Gene Expression Assay	Thermo Fisher	Hs04194366_g1
SP4 Gene Expression Assay	Thermo Fisher	Hs00162095_m1
SP8 Gene Expression Assay	Thermo Fisher	Hs01941366_s1
LINC01162 Gene Expression Assay	Thermo Fisher	Custom made
LARP1B Gene Expression Assay	Thermo Fisher	Hs00292731_m1
IL6 Gene Expression Assay	Thermo Fisher	Hs00174131_m1

(*Continued on next page*)

Continued		
REAGENT or RESOURCE	SOURCE	IDENTIFIER
MRPL19 Reference Gene Expression Assay	Thermo Fisher	Hs00608519_m1
POLR2A Reference Gene Expression Assay	Thermo Fisher	Hs00172187_m1
Recombinant DNA		
Software and Algorithms		
FlowJo v9	Tree Star	https://www.flowjo.com/
QuantaSoft v1.4	Bio-Rad	http://www.bio-rad.com/
R programming language	R Development Core Team, 2015	https://www.R-project.org/
GenomeStudio V2011.1	Illumina team	https://www.illumina.com
PLINK v1.07;v1.9	Purcell et al., 2007	https://www.cog-genomics.org/plink2
SHAPEIT2	Delaneau et al., 2014	http://mathgen.stats.ox.ac.uk/genetics_software/shapeit/shapeit.html
IMPUTE v2.0	Howie et al., 2009	http://mathgen.stats.ox.ac.uk/impute/impute_v2.html
SNPTEST v2	Marchini et al., 2007	https://mathgen.stats.ox.ac.uk/genetics_software/snptest/snptest.html
SNP2HLA v1.0.3	Jia et al., 2013	http://software.broadinstitute.org/mpg/snp2hla/
PRSice v1.25	Euesden et al., 2015	http://prsice.info/
Locuszoom standalone v1.4	Pruim et al., 2010	https://genome.sph.umich.edu/wiki/LocusZoom_Standalone
SWISS v1.0.05b	Developer: Ryan Welch	https://github.com/statgen/swiss
GEMINI v0.20.1	Paila et al., 2013	https://gemini.readthedocs.io/en/latest/
HaploReg v4 tool	Ward and Kellis, 2012	https://pubs.broadinstitute.org/mammals/haploreg/haploreg.php
RegulomeDB v1.1	Boyle et al., 2012	http://www.regulomedb.org/

CONTACT FOR REAGENT AND RESOURCE SHARING

Further information and requests for resources and reagents should be directed to and will be fulfilled by the Lead Contact, Prof. An Goris (an.goris@kuleuven.be). Summary results for suggestive associations are listed in Tables S4 and S6. Summary statistics for all variants are available through the Lead Contact, Prof. An Goris (an.goris@kuleuven.be). Immunological data have been made available previously (Carr et al., 2016). Requests for individual-level genotype data should be directed to and will be fulfilled pending Institutional Review Board approval and accordance with EU General Data Protection Regulation by the Lead Contact, Prof. An Goris (an.goris@kuleuven.be).

EXPERIMENTAL MODEL AND SUBJECT DETAILS

Human subjects

For the GWAS with immune phenotypes, we included n = 502 healthy individuals above 18 years of age, self-reported as healthy and of Caucasian origin from the same cohort in which we previously demonstrated environmental effects on the immune phenotype (Carr et al., 2016). Exclusion criteria were cancer, autoimmunity and gastrointestinal complaints. All individuals gave written informed consent and the study was approved by the Ethics Committee of the University Hospitals Leuven. Blood samples were collected in heparin tubes and rested at 22°C for 4 h before separation of plasma and PBMCs using lymphocyte separation medium (LSM, MP Biomedicals). PBMCs were frozen in 10% DMSO (Sigma) and stored at −80°C for a maximum of 10 weeks. For B cell related expression experiments, we included four healthy donors (2 female, 2 male) for B cell subset RNA expression and 82 multiple sclerosis patients (54 female, 28 male), of which 41 were untreated and 41 were treated with interferon-beta, for total PBMC RNA expression. All individuals gave written informed consent and the study was approved by the Ethics Committee of the University Hospitals Leuven. Blood samples were collected in EDTA tubes and rested at 4°C for a maximum of 2 h before separation of plasma and PBMCs using Lymphoprep (StemCell Technologies, Inc.). PBMCs were frozen in 10% DMSO (Sigma) and stored in liquid nitrogen. For the T helper differentiation experiment, PBMCs from three anonymous blood bank donors were isolated from leukocyte cones using Histopaque-1077 (Sigma).

METHOD DETAILS

Flow cytometry phenotyping

Thawed cells were stained with antibodies as listed in the Key Resource Table. Ki67 and Foxp3 staining was performed after treatment with fixation-permeabilization buffer (eBioscience). Cytokine staining was performed after *ex vivo* stimulation for 5 h in 50 ng/ml PMA (Sigma) and 500 ng/ml ionomycin (Sigma) in the presence of GolgiStop (BD Biosciences). Stimulated cells were surface stained, fixed and permeabilized with Cytofix/Cytoperm (BD), before staining for cytokines. Additional cells were stimulated for 72 h for supernatant assessment by MSD (see below). Data were acquired on a BD FACSCantoII and analyzed with FlowJo (Tree Star).

Serological assessment

Plasma samples collected were stored at −80°C. Circulating levels of B cell activation factor (BAFF) were measured using a human BAFF Quantikine ELISA (R&D Systems). Cytokine plasma concentrations were quantified by electrochemiluminescence immunoassay using the V-Plex human Proinflammatory panel (Meso Scale Discovery). All reagents and standards were provided by each manufacturer. Samples and standards were prepared according to each manufacturer's instructions.

sjKREC and sjTREC levels

Droplet digital PCR (Bio-Rad, Hercules, CA) with DNA detection assays (Thermo Fisher) for sjKREC, sjTREC (both custom made, sequences available on request) and TaqMan Copy Number Reference Assay for human RNase P was performed using 250 ng of restriction digested (EcoRI, New England Biolabs) genomic DNA according to the manufacturer's instructions. Relative quantity of sjKREC and sjTREC per (RNaseP / 2) * 100,000 cells was measured with QuantaSoft v1.4 (Bio-Rad). Correlation among n = 12 duplicate measurements was 0.81 for sjKREC and 0.92 for sjTREC. sjKREC is correlated with B cells ($r^2 = 0.33$), in particular transitional ($r^2 = 0.14$) and naive ($r^2 = 0.22$) B cells, whereas sjTREC is correlated with RTE $CD4^+$ ($r^2 = 0.15$) and $CD8^+$ ($r^2 = 0.13$) T cells.

Immune phenotype data processing

In order to obtain normally distributed data for all traits, a rank-based inverse normal transformation was applied to n = 51 parameters using the rank and qnorm functions in the R statistical software package. For cytokines, levels below the detection threshold were set as equal to the detection threshold. Three cytokines with levels below or equal to the detection threshold in a large subset of individuals (IL-2, IL-4 and IL-12) deviated from a normal distribution even after rank-based inverse normal transformation and were hence transformed into a binary variable representing positive (above detection threshold) or negative (below or equal to detection threshold) measurements. An overview of the 54 immunological parameters is given in Table S1. Sex was taken into account as a covariate in the analyses.

Genotyping

DNA was extracted from total blood using standard methods with an in-house protocol. Concentration was measured with Nanodrop 2000 (Thermo Scientific) and samples were diluted at 100 ng/μl in TE 10/1. All participants were genotyped for 700,078 variants using the Infinium© HTS assay on Global Screening Array bead-chips (Illumina). Genotype calling was done using GenomeStudio V2011.1 software. Genotyping, genotype calling, quality control and imputation were performed together with 217 individuals from the same population included in a separate GWAS for imaging traits (Smets I. et al., manuscript in preparation) to improve imputation accuracy especially for lower allele frequency spectra. Cluster plots were inspected for genome-wide significant directly genotyped lead SNPs or SNPs in LD ($r^2 > 0.3$ and $p < 10^{-4}$) with imputed lead SNPs. "Singleton" imputed lead SNPs (no $r^2 > 0.6$ with other directly genotyped or imputed SNPs) were genotyped using a Taqman assay.

Expression experiments

Naive $CD4^+$ T cells were negatively isolated from PBMCs of three healthy donors using the human Naive $CD4^+$ T Cell Isolation Kit II (Miltenyi Biotec). The purity was measured using flow cytometry (95%–98%). Differentiation of T helper cells into either Th1 or Th17 was carried out using 50 IU/ml IL-2, 1:5 anti-CD2/CD3/CD28 beads (T cell activation/expansion kit, Miltenyi Biotec) and lineage-skewing cytokines: 20 ng/ml IL-12 for Th1 and 80ng/ml IL-1β/IL-6/IL-23 for Th17 cells. IL-12 was purchased from R&D Systems, all other cytokines were purchased from PeproTech. Cells were harvested after 6 days for mRNA. Taqman gene expression assays (Thermo Fisher) were used to measure the mRNA levels of *STON2* (Hs00263833_m1), *SEL1L* (Hs01071406_m1) and *LINC01467* (Hs04403614_m1), with *RPL13A* (Hs04194366_g1) as the reference gene.

PBMCs from four healthy donors were sorted using a B cell panel using the following antibodies: CD24-FITC (BioLegend), IgM-PE (EBioscience), CD14-PE-Cy5.5 (EBioscience), IgD-PE-Cy7 (BioLegend), CD38-APC (EBioscience), CD27-AF-700 (EBioscience), CD19-PE-Cy5 (BioLegend). Cells were sorted into transitional, naive, switched memory and unswitched memory B cells. RNA was extracted from these sorted cells using TRIzol reagent (Thermo Fisher). The same extraction method was used for extracting RNA from PBMCs of 173 of the healthy individuals and 82 multiple sclerosis patients. RNA was reverse transcribed using the High-Capacity cDNA Reverse Transcription Kit (Thermo Fisher). Droplet digital PCR (Bio-Rad) was performed using up to 50 ng cDNA according to the manufacturer's instructions with predesigned gene expression assays (Thermo Fisher) for *SP4* (Hs00162095_m1), *SP8* (Hs01941366_s1), *LARP1B* (Hs00292731_m1) and *IL6* (Hs00174131_m1), a custom Taqman gene

expression assay for *LINC01162*, and assays for reference genes *POLR2A* (Hs00172187_m1) and *MRPL19* (Hs00608519_m1). Relative quantity of *SP4*, *SP8* and *LINC01162* versus *POLR2A* and of *LARP1B* and *IL6* versus *MRPL19* was measured with QuantaSoft v1.4 (Bio-Rad).

QUANTIFICATION AND STATISTICAL ANALYSIS

Sample size and power

The sample size of n = 502 healthy individuals provided 80% power to detect at genome-wide significance level ($p < 5 \times 10^{-8}$) genetic variants explaining $\geq$ 7.8% of variance in a trait, in line with realistic effect sizes previously observed (Orrù et al., 2013).

Genotyping quality control

Sample QC was performed using PLINK v1.07 (see also Figure S1). Gender was verified using the mean homozygosity rate across X chromosome markers. Samples with genotype call rate < 98% or excess heterozygosity (> 5 standard deviations from the sample mean) were excluded based on the set of variants meeting minor allele frequency (MAF) $\geq$ 5%, genotyping success rate $\geq$ 98% and Hardy-Weinberg $p > 10^{-6}$ (n = 3, Figure S1A). The biological relationship of all individuals was verified based on pairwise identity by descent (IBD), and identical or related individuals were removed (n = 8, Figure S1B). For this analysis, regions of extended LD (Price et al., 2008; Weale, 2010) were removed, and the dataset was pruned so that no pair of SNPs within a given window of 50kb is correlated ($r^2 > 0.2$). For each pair showing cryptic relatedness (defined as IBD > 0.1875), the individual with the lower genotyping call rate was removed from further analysis. In addition, ethnic outliers from the European cluster as assessed by principle component analysis (PCA) combined with 1000 Genomes populations Phase 1 (1000 Genomes Project Consortium, 2012) were removed (n = 2, Figure S1C). Both IBD estimation and clustering for population stratification were based on a pruned set of independent markers (defined by pairwise $r^2 < 0.2$). A total of n = 489 healthy controls remained in the analysis.

Variant QC was performed in the cleaned sample set. Variants with minor allele frequency (MAF) < 1%, call rate < 98% for common variants (MAF > 5%) or < 99% for low frequency variants (1% $\leq$ MAF $\leq$ 5%) or significant deviation from Hardy-Weinberg equilibrium (HWE) ($p < 10^{-6}$) were excluded. A total of 500,542 SNPs remained in the analysis. Alleles were aligned to b37 forward strand alleles to be in accordance with the imputation reference panel of haplotypes (strand file used: http://www.well.ox.ac.uk/~wrayner/strand/).

Genotype imputation

Before imputation genotypes were pre-phased using SHAPEIT2 (O'Connell et al., 2014) with the European 1000 Genomes October 2014 haplotypes [Phase 3 integrated variant set release in NCBI build 37 (hg19) coordinates] as a reference panel (http://www.internationalgenome.org/). For pre-phasing, default parameters were used for the autosomes and pseudoautosomal regions of the X chromosome, whereas the '–chrX' flag was used for the non-pseudoautosomal region of the X chromosome.

The imputation into the estimated haplotypes was performed using the IMPUTE v2.0 software package (Howie et al., 2009) and all 1000 Genomes October 2014 haplotypes [Phase 3 integrated variant set release in NCBI build 37 (hg19) coordinates] (http://www.internationalgenome.org/). All samples were pre-phased and imputed in a single batch, to avoid batch effects attributable to the imputation process. To make computation feasible during imputation, each chromosome was split into 5-Mb chunks with default parameters for the autosomal and pseudoautosomal regions and the '-chrX' flag for the non-pseudoautosomal regions of the X chromosome.

The MHC region on chromosome 6 (chr 6:29494896-33160424) was extracted from the post-QC directly genotyped dataset. Classical Human Leukocyte Antigen (HLA) alleles, amino acid polymorphisms and SNPs in the MHC region were imputed with SNP2HLA v1.0.3 and the T1DGC reference panel (build 37) (Jia et al., 2013). Imputed HLA variants were merged with directly genotyped variants and IMPUTE v2.0 imputed variants in the region leading to 132,064 unique variants, including 424 classical HLA alleles and 1,276 HLA amino acid changes.

GWAS analysis

A genome-wide association analysis of directly genotyped and imputed variants was performed for each trait. An additive genetic model was fitted at each variant with gender as covariate using SNPTESTv2 software with the frequentist test and the expectation-maximization method (as implemented in SNPTEST) to account for genotype uncertainty in the regression analyses of the imputed SNPs and especially low frequency ones (Marchini et al., 2007). The Phase 3 haplotypes in SNPTESTv2 contain multi-allelic variants, and SNPTEST processes these variants to create multiple bi-allelic variants. For example, a tri-allelic SNP with 3 alleles (REF, ALT1, ALT2) will have been recoded as two bi-allelic SNPs, the first one with alleles REF and ALT1, and the second SNP with alleles REF and ALT2. For markers on the X chromosome, the association test was performed assuming a standard model of complete X inactivation and an equal effect size in men and women. In this model, male genotypes are coded as 0/1 and female genotypes as 0/0.5/1.

The association results were quality controlled by removing the following variants: MAF < 1%, minor allele count $\leq$ 6 and imputation quality score (SNPTEST Proper_INFO) < 0.4. Results were obtained for a maximum of 10,246,977 autosomal variants, including n = 6,994,434 common variants (n = 6,086,969 SNPs and n = 907,465 indels) and n = 3,252,543 less common variants

(n = 2,962,706 SNPs and n = 289,837 indels) (see also Figure S2). The genomic control inflation factor (λ_{GC}) had a median of 1.012 (range 0.995 - 1.033) suggesting population stratification had limited influence on the test statistical distribution. Associations were considered genome-wide significant for $p < 5 \times 10^{-8}$ and suggestive for $p < 10^{-4}$. The lead SNP was identified as the SNP with smallest P value in a 1-Mb region.

Chromosome X results with MAF $\geq$ 0.01, MAC > 6 and INFO $\geq$ 0.4 were obtained for up to 353,613 variants including n = 224,865 common variants (n = 190,475 SNPs and n = 34,390 indels) and n = 128,748 less common variants (n = 113,977 SNPs and n = 14,771 indels).

Percentage of the variance explained for each genome-wide significant variant was calculated on the basis of a generalized linear model in PRSice v1.25 as the r^2 of the full model (genetic variant and gender) minus r^2 of the baseline model (only including gender).

Conditional analysis and LD pruning

Conditional analyses were performed using SNPTESTv2 for SNPs in a 2-Mb region around each genome-wide significant lead SNP in a linear regression additive model conditioning on the lead SNP. Quality control was applied as above.

Regional association plots were generated using the stand-alone LocusZoom version 1.4 with 1000 Genomes genotypes (1000G_Nov2014, EUR) build hg19 as LD resource and refflat for gene annotation.

LD-based clumping was performed for all autosomal variants reaching suggestive association ($p < 10^{-4}$) per trait and across traits using SWISS version 1.0.05b. Variants in LD ($r^2 > 0.1$ based on the build-in 1000G_2014-11_EUR) within 1-Mb from the most significantly associated variant were removed so that independent variants remain. For N = 49 variants for which no LD information was available in the 1000G EUR dataset, the 1000G ALL dataset was used instead.

Variant annotation

Variants were annotated for location, functional consequences, and predicted characteristics with GEMINI (Paila et al., 2013) version 0.20.1 with assembly GRCh37.p13, RefSeq 87_GRCh37, dbSNP 137, and VEP v.87. HaploReg v4 tool was used to examine whether variants or variants in LD ($r^2 > 0.8$) are coding, conserved (as predicted as such by both GERP and SiPhy scores), or overlapping with quantitative trait loci. Additionally, ExSNP integrated eQTL database of 16 publicly available human QTL sources (Yu et al., 2016) and QTL studies on relevant immunological cell subsets (listed in Lotta et al. (Lotta et al., 2017)) were queried. For variants listed in the main tables, the LD-based query (variant or variant with $r^2 > 0.8$) in ExSNP was used; for the full list of suggestive variants the full ExSNP dataset was downloaded and cross-linked for overlap. For splicing QTLs in whole blood, a published dataset was used (Zhang et al., 2015b). Disruption of a regulatory motif was evaluated using RegulomeDB v1.1.

The SWISS software version 1.0.05b was used to ascertain whether there is LD ($r^2 > 0.8$ based on the build-in 1000G_2014-11_EUR) between suggestive variants in our study and known associations reaching genome-wide significance with other traits in the build-in EBI GWAS catalog GRCh37p13.

Expression analysis

For *in vitro* differentiated Th1 and Th17 cells from each of three donors, qPCR was performed in triplicate, the average of which is plotted. Two-tailed paired t test was used for comparison of the log-transformed relative quantity (RQ) in Th17 versus Th1 differentiated cells, with the average RQ in Th1 set as equal to 1. For sjKREC and log-transformed *LARP1B* relative quantity in 82 multiple sclerosis patients, linear regression with covariates age, gender and treatment was applied.

DATA AND SOFTWARE AVAILABILITY

Immunological data have been made available previously (Carr et al., 2016). Summary results for suggestive associations are listed in the Tables S4 and S6. Summary statistics for all variants are available through the Lead Contact, Prof. An Goris (an.goris@kuleuven.be).

The following publicly available software packages were used for the analyses: Plink v1.07 (Purcell et al., 2007), SHAPEIT (O'Connell et al., 2014), IMPUTE v2.0 (Howie et al., 2009), SNPTESTv2 (Marchini et al., 2007), SNP2HLA v1.0.3 (Jia et al., 2013), LocusZoom standalone, SWISS version 1.0.05b, GEMINI version 0.20.1. (Paila et al., 2013), Haploreg v4.1, and RegulomeDB v1.1. The following publicly available datasets were used for the analyses: strand file for Illumina GSA array: http://www.well.ox.ac.uk/~wrayner/strand/; 1000 Genomes reference datasets: http://www.internationalgenome.org/; the T1DGC reference panel for the HLA region (build 37) (Jia et al., 2013); the ExSNP dataset (Yu et al., 2016); and the EBI GWAS catalog GRCh37p13.

Cell Reports, Volume 25

Supplemental Information

Genetic Architecture

of Adaptive Immune System

Identifies Key Immune Regulators

Vasiliki Lagou, Josselyn E. Garcia-Perez, Ide Smets, Lies Van Horebeek, Marijne Vandebergh, Liye Chen, Klara Mallants, Teresa Prezzemolo, Kelly Hilven, Stephanie Humblet-Baron, Matthieu Moisse, Philip Van Damme, Guy Boeckxstaens, Paul Bowness, Bénédicte Dubois, James Dooley, Adrian Liston, and An Goris

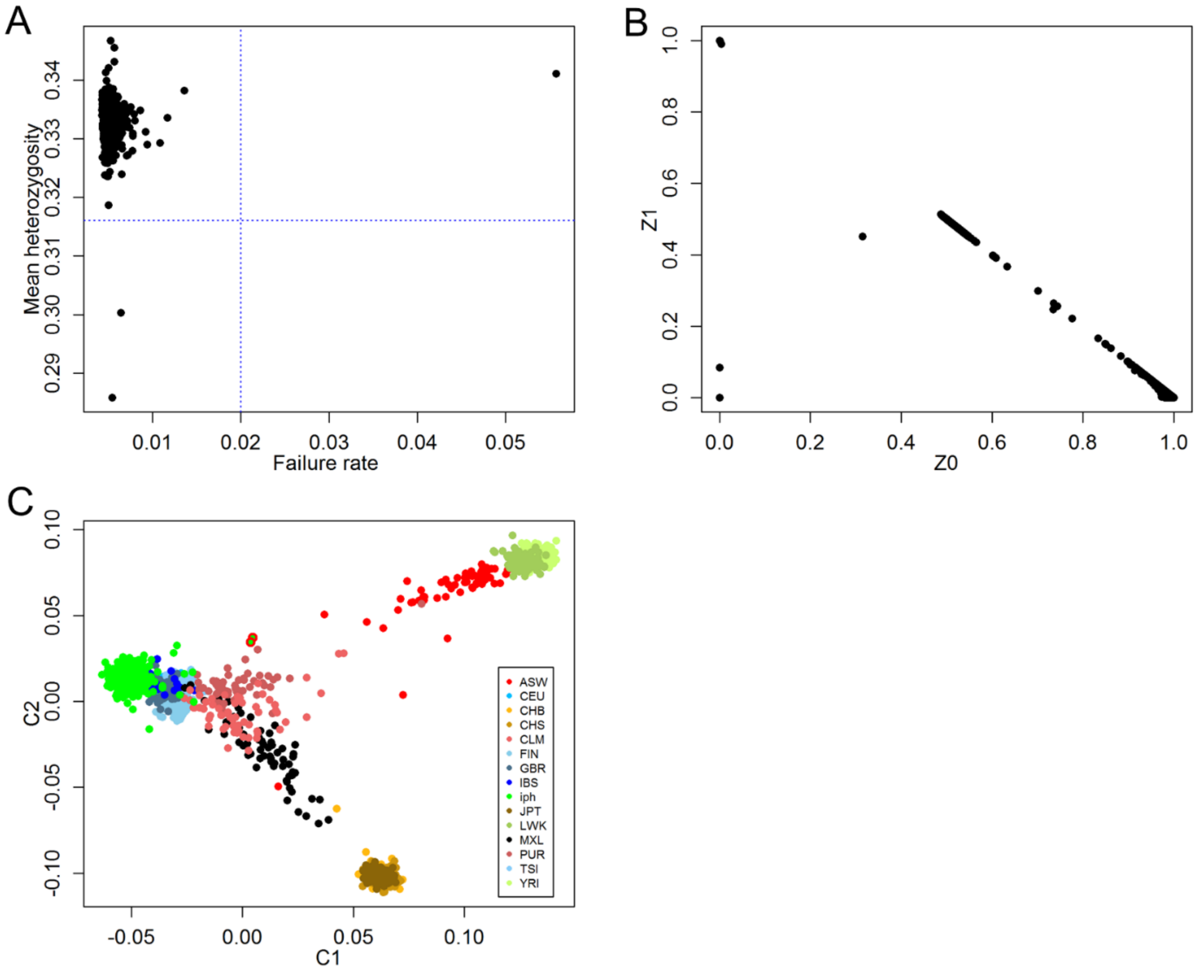

Figure S1. Sample quality control (QC) steps. Related to STAR Methods.
n = 502 samples were genotyped, and quality control (QC) was performed as described in the STAR Methods. n = 13 samples failed QC because of the following reasons: genotyping failure rate and heterozygosity (**A.,** n = 3), duplicates and relatedness (**B.**, n = 8) and ethnicity (**C.**, n = 2). n = 489 samples passed QC and were used as input for imputation as described in the STAR Methods.

A. Genotyping failure rate versus heterozygosity.
Sample QC was based on the set of variants meeting minor allele frequency (MAF) ≥ 5%, genotyping success rate ≥ 98% and Hardy-Weinberg P > 10^{-6}. Three samples with genotype call rate < 98% (1 sample) or excess heterozygosity (> 5 standard deviations from the mean) (2 samples) failed QC.

B. Identity-by-descent (IBD) analysis.
For the IBD analysis, we removed regions of extended linkage disequilibrium (LD) from the dataset and pruned remaining regions so that no pair of variants within a given window of 50 kb is correlated ($r^2 > 0.2$). The X- and Y- axis represent Z0 (P(IBD = 0)) and Z1 (P(IBD = 1)), the probabilities of sharing 0 or 1 alleles identical-by-descent. Four samples were identified as duplicates (expected Z0 = Z1 = 0), three samples as parent-offspring (Z0 = 0 and Z1 = 1), and one sample as half-sibling (expected Z0 = Z1 = 0.5).

C. Principal component analysis.
The first two principal components (C1 and C2) are calculated for the current dataset (Iph, bright green) together with 1000G Phase 1 data for the following populations: ASW = Americans of African Ancestry in SW USA; CEU = Utah Residents (CEPH) with Northern and Western European Ancestry; CHB = Han Chinese in Bejing, China; CHS = Southern Han Chinese; CLM = Colombians from Medellin, Colombia; FIN = Finnish in Finland; GBR = British in England and Scotland; IBS = Iberian Population in Spain; JPT = Japanese in Tokyo, Japan; LWK = Luhya in Webuye, Kenya; MXL = Mexican Ancestry from Los Angeles USA; PUR = Puerto Ricans from Puerto Rico; TSI = Toscani in Italia; YRI = Yoruba in Ibadan, Nigeria). Two individuals (green encircled with orange) were removed as outliers from the European cluster. Further inspection revealed these individuals as being of North-African descent.

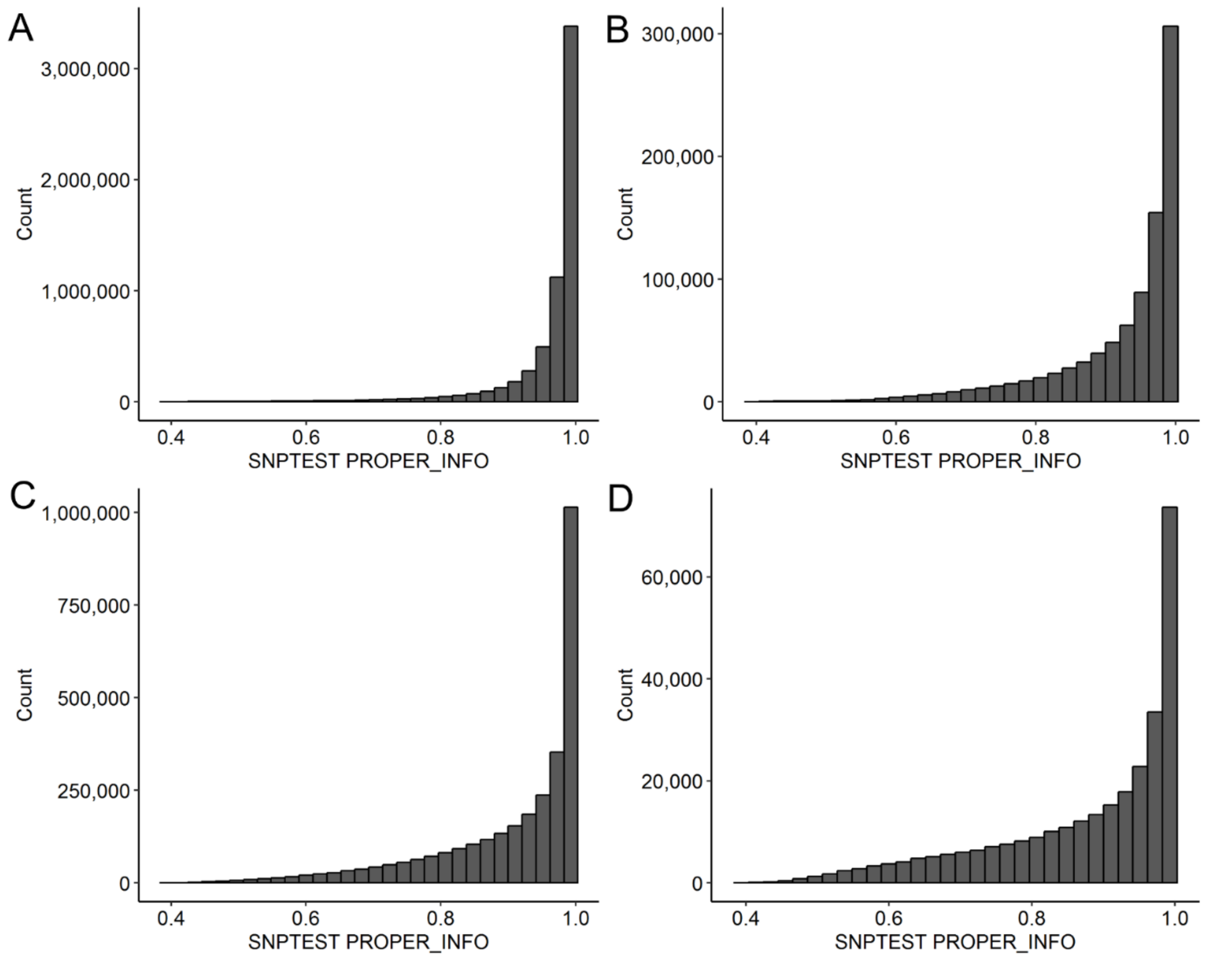

Figure S2. Imputation quality for genetic variants included upon QC. Related to STAR Methods.
Histograms of the imputation quality scores (average SNPTEST PROPER_INFO across association results for n = 54 traits) for the following categories amongst 10,246,977 autosomal variants:
A. Common (MAF > 5%) single-nucleotide variants (n = 6,086,969)
B. Common (MAF > 5%) indels/structural variants (n = 907,465)
C. Less common (1% ≤ MAF ≤5%) single-nucleotide variants (n = 2,962,706)
D. Less common (1% ≤ MAF ≤ 5%) indels/structural variants (n = 289,837)
